# Optimizing Loop Diuretic Treatment for Mortality Reduction in Patients With Acute Dyspnea Using a Practical Offline Reinforcement Learning Pipeline for Health Care: Retrospective Single-Center Simulation Study

**DOI:** 10.2196/69145

**Published:** 2025-10-10

**Authors:** Jung Min Lee, Shengpu Tang, Michael Sjoding, Jenna Wiens

**Affiliations:** 1 Division of Computer Science and Engineering, College of Engineering, University of Michigan, 2260 Hayward St, Ann Arbor, MI, 48109, United States, 1 7346474832; 2 Department of Computer Science, Emory College of Arts and Sciences, Emory University, Atlanta, GA, United States; 3 Division of Pulmonary and Critical Care Medicine, Michigan Medicine, University of Michigan, Ann Arbor, MI, United States

**Keywords:** reinforcement learning, artificial intelligence, loop diuretic, treatment recommendation, treatment selection, clinical decision support, dynamic treatment regime

## Abstract

**Background:**

Offline reinforcement learning (RL) has been increasingly applied to clinical decision-making problems. However, due to the lack of a standardized pipeline, prior work often relied on strategies that may lead to overfitted policies and inaccurate evaluations.

**Objective:**

In this work, we present a practical pipeline—Pipeline for Learning Robust Policies in Reinforcement Learning (PROP-RL)—designed to improve robustness and minimize disruption to clinical workflow. We demonstrate its efficacy in the context of learning treatment policies for administering loop diuretics in hospitalized patients.

**Methods:**

Our cohort included adult inpatients admitted to the emergency department at Michigan Medicine between 2015 and 2019 who required supplemental oxygen. We modeled the management of loop diuretics as an offline RL problem using a discrete state space based on features extracted from electronic health records, a binary action space corresponding to the daily use of loop diuretics, and a reward function based on in-hospital mortality. The policy was trained on data from 2015 to 2018 and evaluated on a held-out set of hospitalizations from 2019, in terms of estimated reduction in mortality compared to clinician behavior.

**Results:**

The final study cohort included 36,570 hospitalizations. The learned treatment policy was based on 60 states: the policy deferred to clinicians in 36 states, recommended the majority action in 22 states, and diverged significantly from clinician behavior in 2 of the states. Among the cases where the policy meaningfully diverged from the behavior policy, the learned policy was estimated to significantly reduce the mortality rate from 3.8% to 2.2% by 1.6% (95% CI 0.4‐2.7; *P*=.006).

**Conclusions:**

We applied our pipeline to the clinical problem of loop diuretic treatment, highlighting the importance of robust state representation and thoughtful policy selection and evaluation. Our work reveals areas of potential improvement in current clinical care for loop diuretics and serves as a blueprint for using offline RL for sequential treatment selection in clinical settings.

## Introduction

Reinforcement learning (RL) is a branch of artificial intelligence that, through interactions with an environment, learns the optimal sequence of actions that will maximize a desired outcome [1]. RL methods are especially well suited to tackle problems that require sequential decision-making where the rewards are delayed. This makes it an attractive solution for learning dynamic treatment policies in health care problems (eg, sepsis [2], diabetes [3], and hypotension [4]) where decisions are made sequentially over a prolonged period of time and the outcome (eg, in-hospital mortality) is observed at a later time point. Due to safety and ethical concerns, training and evaluation of RL policies in this domain often rely on a fixed set of historical data and require the use of offline RL algorithms [5].

However, effectively applying offline RL poses several challenges. First, deriving a robust and informative state representation from high-dimensional health features can be challenging, especially with limited data. Second, the performance of offline RL algorithms is sensitive to hyperparameters [6-8], often leading to policies that perform well during development but fail once deployed. Yet a standardized approach for hyperparameter selection has not been established for offline RL. Third, the learned policy may differ substantially from current clinician behavior, resulting in low confidence in evaluation results and potential disruption to clinical workflows [9]. While some of these issues have been solved in isolation [10-12], there is a notable absence of a standard pipeline for applying offline RL, comparable to the training-validation framework in supervised learning, that integrates these individual solutions. We address this gap by presenting a pipeline (Pipeline for Learning Robust Policies in Reinforcement Learning; PROP-RL) along with a codebase for applying offline RL to health care settings, and demonstrate its efficacy by applying it to the problem of learning treatment policies for loop diuretics.

Loop diuretics are one of the most commonly prescribed medications in hospitals and are used to control volume and edema in the body by increasing urinary sodium and water excretion [13]. They are used to treat patients with acute shortness of breath from fluid accumulation in their lungs, typically associated with conditions such as congestive heart failure or acute pulmonary edema [14]. There remains substantial uncertainty and variability regarding when to start and stop loop diuretics [15,16]. This uncertainty leads to inadequate use of loop diuretics, which has been associated with worse clinical outcomes, including higher rates of acute kidney injury and electrolyte disturbances [17,18].

In this paper, we apply offline RL to learn a loop diuretics treatment policy—designed to aid health care professionals—from electronic health records (EHRs) of hospitalized patients at a large academic hospital. In doing so, we establish a pipeline—PROP-RL—for applying offline RL in health care settings that incorporates state representation learning, hyperparameter selection, and modification of the learned policy to minimize disruption to existing workflows. We demonstrate the effectiveness of PROP-RL through off-policy evaluation (OPE) and ablation studies [19].

## Methods

### Study Cohort

We included adult patients (≥18 years) admitted to the hospital through the emergency department at Michigan Medicine during the years 2015‐2019, who required any amount of supplemental oxygen support during the first 24 hours of admission. Patients who underwent surgery within 24 hours of admission were excluded as the supplemental oxygen support provided may not be due to a primary respiratory condition (Section A1 in Multimedia Appendix 1). The cohort was split into a development set and a held-out test set consisting of data from patients admitted in 2015‐2018 and 2019, respectively.

### Data Preprocessing

**Figure 1.**
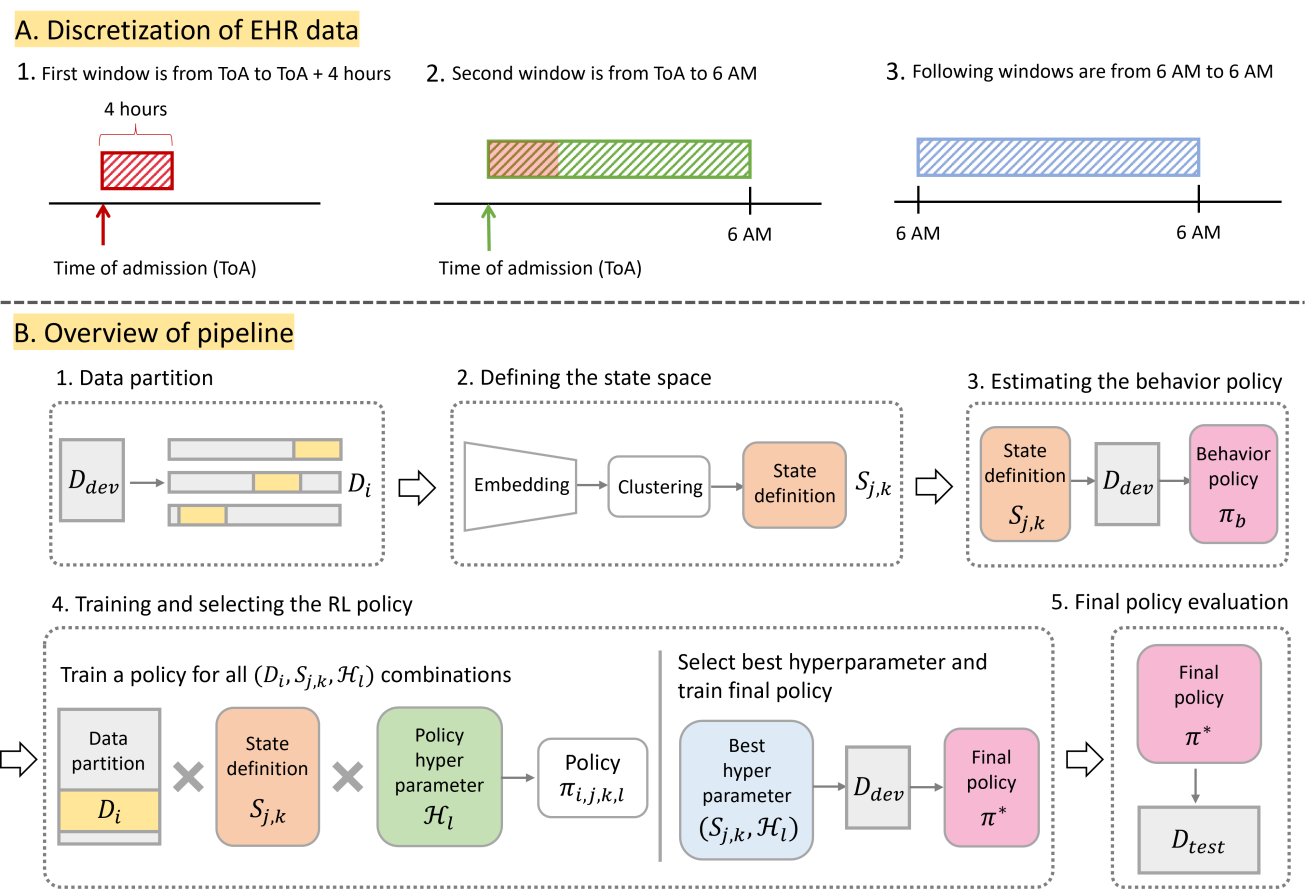
(A) Diagram of the windowing rule for hospitalizations. (B) Overview of pipeline. (1) Data partition: the development data *D*_*dev*_ are partitioned in multiple ways to create the data partitions *D*_*i*_, *i* ∈ {1...10}. (2) Defining the state space: a set of candidate discrete state definitions, characterized by the data partition *D*_*j*_ used to derive the state definition and the number of states *k*, is generated by learning a lower-dimensional representation of the features and clustering them. (3) Estimating the behavior policy: the behavior policy *π_b_* is estimated from the development state using each state definition *S_j,k_*. While *π_b_* is dependent on *S_j,k_*, for simplicity, we refer to the behavior policy as *π_b_* in general. (4) Training and selecting the RL policy: a policy *π_i, j, k, l_* is trained for each possible hyperparameter combination across all data partitions. The best hyperparameter is used to train the final policy π^*^ on the entire development set. (5) Final policy evaluation: π^*^ is evaluated on the test set *D_test_*. EHR: electronic health record; RL: reinforcement learning.

To formulate the management of loop diuretics as an RL problem with discrete time steps, we split the hospitalization data into chronological windows. With the exception of the first and second windows, all windows were 24 hours long, starting and ending at 6 AM (Figure 1A; Section A2 in Multimedia Appendix 1). In each window, medication records were analyzed to determine whether an oral or intravenous loop diuretic was administered. We assumed all treatment decisions made within a window were based on the patient’s state in the previous window. A 6 AM cutoff time was chosen as most clinical rounds (where decisions are made) occur immediately after this point. Analysis was constrained to the first 8 days of hospitalization.

For each window, EHR features including age, vital sign measurements, laboratory test results, medications, fluid input and output, and Sequential Organ Failure Assessment (SOFA) scores were extracted (Section A3 in Multimedia Appendix 1). These features capture the patient’s most recent health state as well as past treatments, which are necessary for determining future treatments. We used the Flexible Data-Driven Pipeline (FIDDLE) software to convert these into 243-dimensional feature vectors (Section A2, A3, and A16 in Multimedia Appendix 1) [20].

### Model Development and Evaluation

#### Overview

We modeled the patient environment as a Markov decision process (MDP) defined by (*S*, *A*, *P*, *R*, *γ*). *S* and *A* represent the state and action spaces. Given a hospitalization, *s_t_*∈*S* represents the patient’s health on day *t* and *a_t_* ∈ *A* is the treatment decision made based on $s_{t}$. *P* (*s*_*t*+1_|*s*_*t*_, *a*_*t*_) is the transition function, *R*(*s_t_*) = *r_t_* is the reward function, and *γ* ∈ [0, 1] is the discount factor. The discrete state space *S* was defined by clustering the EHR features in a learned embedding space. The action space *A* = {0, 1} was defined to encode binary treatment decisions, corresponding to whether the patient received loop diuretics (Section A4 in Multimedia Appendix 1). All intermediate rewards were set to 0, and a terminal reward was given when the patient’s hospitalization ended or reached 8 days (whichever is earlier). The terminal reward was 100 if the patient was discharged alive and –100 if the patient died. Our objective was to learn a policy *π*: *S*×*A* → [0, 1] which maps *s*_*t*_ to a probability distribution over *a*_*t*_, in order to maximize the expected cumulative reward ${J(\pi )=E}_{\pi }\left[\sum_{t=0}^{9}\gamma^{t}R(s_{t})\right]$ where *γ*=0.99. This roughly corresponds to an objective that focuses on minimizing the overall mortality rate.

PROP-RL consists of the following 5 steps (Figure 1B): (1) data partition, (2) defining the state space, (3) estimating the behavior policy, (4) training and selecting the RL policy, and (5) final policy evaluation.

#### Step 1. Data Partition

We created 10 partitions of the development set by randomly assigning each hospitalization to either the training or validation split. These partitions were used for steps (2) and (4).

#### Step 2. Defining the State Space

We used a data-driven approach to establish state definitions. For each data partition, a function mapping the 243-dimensional feature space to a discrete state space was learned by training a neural network embedding model and applying ensemble *k*-means clustering in the embedding space (Section A5 in Multimedia Appendix 1) [21,22]. *k*, the size of the discrete state space, was a hyperparameter that varied from {20,40,...,160} (Section A6 in Multimedia Appendix 1). The state definition itself was treated as a hyperparameter.

Prior to policy learning, the state definitions were evaluated for generalizability and informativeness. We verified that each hospitalization transitioned across multiple different states and that the state distribution was not heavily skewed toward a few specific states. Failing to meet both criteria implies an overfitted state definition unlikely to generalize to new patients. Second, to ensure the embeddings captured important information, we conducted a principal component analysis of the cluster centers. We visualized the distribution of the cluster centers using the average and SD of the mortality rates, SOFA scores, and clinicians’ previous and next actions among the windows belonging to each state.

#### Step 3. Estimating the Behavior Policy

We estimated the behavior policy by computing the average observed action for each state within the development set. This is a stochastic policy that maps each state to a probability over the binary actions. To further validate the state definitions, we performed 2 evaluations using the estimated behavior policy. First, we compared the estimated mortality rate of the behavior policy on the held-out test set to the true mortality rate. Significant differences in these values would either indicate a significant change in clinicians’ behavior between the 2 datasets, or the state definitions’ inability to encode the behavior policy. Second, we investigated whether the estimated behavior policy aligns with clinical understanding by visualizing trends in the behavior policy with respect to key features of the states (Section A7 in Multimedia Appendix 1).

#### Step 4. Training and Selecting the RL Policy

After learning the transition matrix from the training data, we learned the optimal policy using a modified version of value iteration with 2 offline RL constraints: batch-constrained Q-learning (BCQ) and pessimistic Markov decision process (pMDP) [1,23,24]. These constraints mitigate extrapolation error, which refers to inaccurate value estimations for state-action pairs that were rarely or never observed during training [23]. In brief, BCQ constrains the policy to avoid actions unlikely to be selected by the behavior policy, and pMDP encourages the policy to avoid areas in the state-action space with high uncertainty (Section A8 in Multimedia Appendix 1). Both BCQ and pMDP have additional hyperparameters.

Recent work found hyperparameter selection in offline RL to be sensitive to the partitioning of the dataset [7]. To mitigate this, we use the Split-Select-Retrain (SSR) pipeline that selects the optimal hyperparameters by aggregating validation performance over multiple partitions of the development set [7]. The final policy is then learned from the entire development set using the selected optimal hyperparameters. We leveraged the same 10 partitions (train and validation split) described in step (1) (Section A9 in Multimedia Appendix 1).

Performance was measured using the OPE method weighted importance sampling (WIS), known for its simplicity and reliance on relatively few assumptions [25]. WIS is a biased but consistent estimator, with estimates converging to the true value as sample size increases. WIS estimates both the performance and effective sample size (ESS) of the policy, which is a measure of confidence in the performance estimate [26]. ESS values closer to the size of the dataset used for evaluation (ie, validation set) indicate higher confidence in the performance estimate. For the main analysis, we focus on WIS, but for robustness, we also consider 3 additional OPE methods: fitted Q evaluation, approximate model, and weighted doubly robust estimates (Section A10 in Multimedia Appendix 1 for methodological details). *P* values are estimated by a one-sided bootstrap resampling test [27].

In order to minimize disruption to clinical workflow without sacrificing policy performance, we modified the learned policy prior to evaluation by identifying “unimportant states,” inspired by Shen et al [28]. In unimportant states, no action can significantly impact the outcome. Our policies deferred to clinicians’ decisions in unimportant states, thus minimizing the amount of potential deviation from clinician behavior. The threshold used to determine unimportance was considered a hyperparameter (Section A11 and A12 in Multimedia Appendix 1).

#### Step 5. Final Policy Evaluation

The final policy was evaluated on the held-out test set using WIS. Improvement in performance compared to the behavior policy was measured across 1000 bootstrapped samples in terms of expected cumulative reward and mortality. The level of disagreement between the average clinician and the final learned policy was compared to the level of disagreement among clinicians (Section A13 in Multimedia Appendix 1).

To understand how the learned policy differs from the behavior policy, we focused on “divergent” states where the action recommended by the learned policy diverged from the majority action of the behavior policy. The learned policy was then evaluated on a subset of the cohort where the patient’s hospitalization included divergent states. We further characterized these states by comparing the average values of their key features to those of the general population (Section A14 in Multimedia Appendix 1).

### Ablation Studies of Pipeline

Our pipeline included 3 key elements designed to improve the robustness of the learned policy: (1) using unimportant states to “relax” the learned policy, (2) evaluation across multiple data partitions (SSR), and (3) treating state definitions as a hyperparameter. To demonstrate the effect of each element on the robustness of the learned policy, we conducted an ablation study by selectively removing each component from the pipeline. As a proxy for measuring robustness, we looked at the worst-case OPE performance of the learned policies to establish an empirical lower bound.

### Ethical Considerations

This study was approved by the Institutional Review Board at the University of Michigan Medical School (HUM00141899) with a waiver of informed consent among study patients. All data collected were deidentified and were accessed via a secure cloud storage platform and a secured, Health Insurance Portability and Accountability Act (HIPAA)–compliant server. Participants were not compensated for the use of their data in this study. The study followed the TRIPOD+AI (Transparent Reporting of a Multivariable Prediction Model for Individual Prognosis or Diagnosis+Artificial Intelligence) reporting guideline [29] (Checklist 1). As this study was retrospective in nature, no formal study protocol was developed and the study was not registered. No patients or the public were involved in any aspect of this study.

## Results

### Study Cohort and Patient Characteristics

The initial cohort consisted of 57,907 hospitalizations. We removed cases where supplemental oxygen was not given within 24 hours (n=14,902), patients were moved to surgery within 24 hours (n=6283), and hospitalizations lasting shorter than 2 windows (n=152). The final study population contained 23,945 unique patients and 36,570 unique hospitalizations divided temporally by admission year into the development (n=29,765; 2015‐2018) and test set (n=6805; 2019) (Table 1; Section A1 in Multimedia Appendix 1). The mortality rate of the entire cohort was 5.4% (1978/36,570), and 5.2% (1555/29,765) and 6.2% (423/6805) for the development and test set, respectively.

**Table 1.** Cohort characteristics. Values are numbers (percentages) unless stated otherwise.

Cohort	Overall (2015‐2019)	Development set (2015‐2018)	Test set (2019)
Hospitalizations, n	36,570	29,765	6805
Age (years), median (IQR)	64 (53‐74)	64 (52‐74)	65 (54‐75)
Age range (years), n (%)			
18‐25	1142 (3.1)	1010 (3.4)	124 (1.8)
26‐45	4747 (13.0)	3889 (13.1)	858 (12.6)
46‐65	13,770 (37.7)	11,266 (37.8)	2504 (36.8)
66‐85	14,317 (39.1)	11,477 (38.6)	2840 (41.7)
>85	2594 (7.1)	2123 (7.1)	471 (6.9)
Sex, n (%)			
Female	17,364 (47.5)	14,241 (47.8)	3123 (45.9)
Male	19,206 (52.5)	15,524 (52.2)	3682 (54.1)
Self-reported race, n (%)			
White or Caucasian	30,529 (83.5)	24,853 (83.5)	5676 (83.4)
Black or African American	4295 (11.7)	3503 (11.8)	792 (11.7)
Asian	642 (1.8)	516 (1.7)	126 (1.8)
American Indian or Alaska Native	141 (0.4)	115 (0.4)	26 (0.4)
Native Hawaiian or Other Pacific Islander	27 (0.1)	21 (0.1)	6 (0.1)
Other	659 (1.8)	546 (1.8)	113 (1.7)
Unknown	209 (0.6)	160 (0.5)	49 (0.7)
Patient refused	68 (0.2)	51 (0.2)	17 (0.2)
Hospitalization outcome, n (%)			
Alive	34,592 (94.6)	28,210 (94.8)	6382 (93.8)
Death	1978 (5.4)	1555 (5.2)	423 (6.2)
Length of stay (days), median (IQR)	6 (4-9)	6 (4-9)	6 (4-10)
Length of stay (days), n (%)			
1‐3	8216 (22.5)	6939 (23.3)	1277 (18.8)
4‐5	9446 (25.8)	7736 (26.0)	1710 (25.1)
6‐9	10,027 (27.4)	8063 (27.1)	1964 (28.9)
10‐15	5136 (14.0)	4046 (13.6)	1090 (16.0)
>15	3745 (10.2)	2981 (10.0)	764 (11.2)

### Evaluation of State Definitions

**Figure 2.**
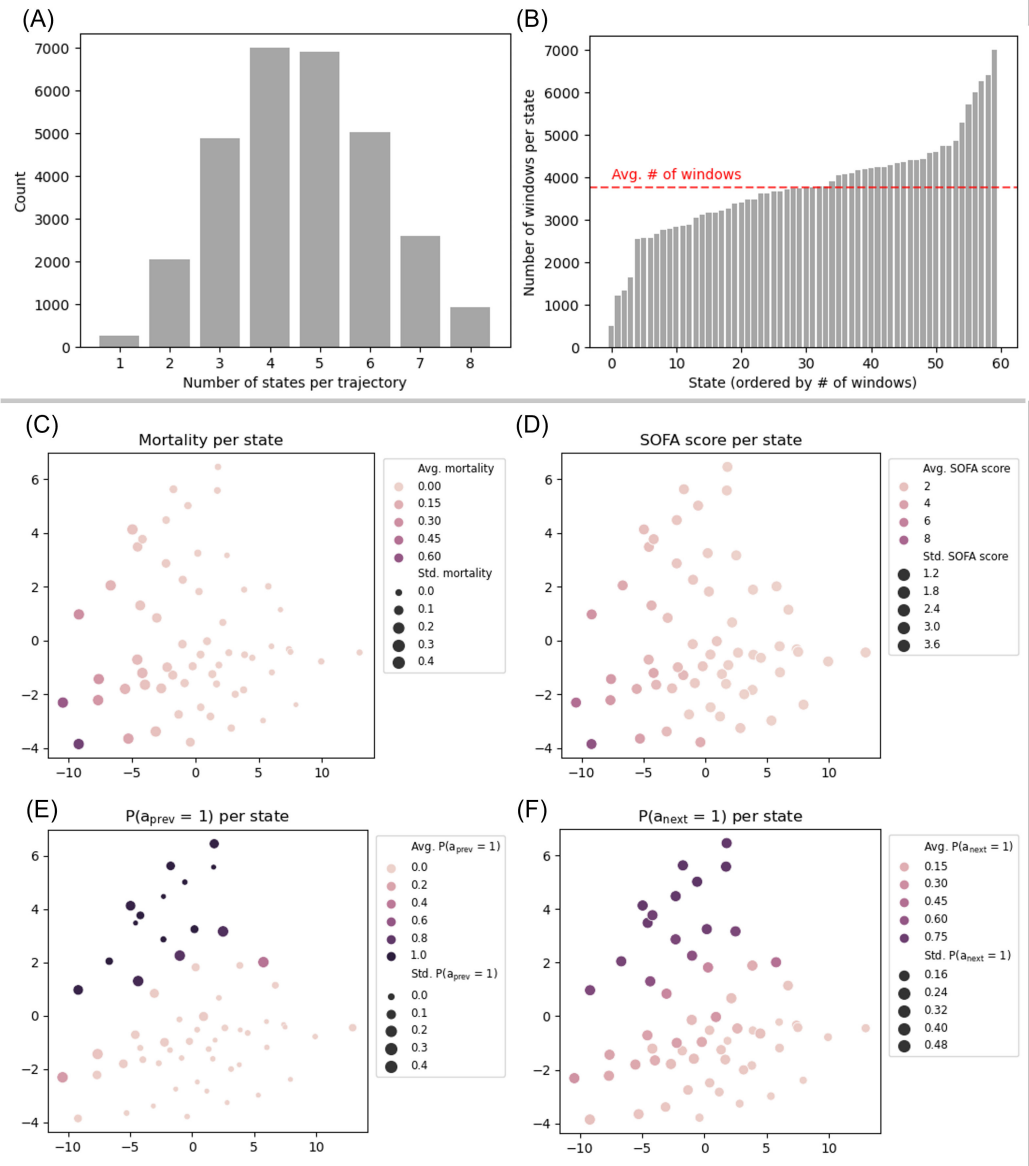
Sanity checks for the state definitions on the development set. The first row shows histograms depicting (A) number of states in each trajectory and (B) the number of windows in each state. The second and third rows show principal component analysis of the representations of the cluster centers that define each state. The hue and size of each dot represent the average and SD of the feature value of all samples in that state. The features are (C) mortality rate, (D) SOFA score, (E) whether loop diuretics were administered in the past 24 hours, and (F) whether clinicians chose to administer loop diuretics. SOFA: Sequential Organ Failure Assessment.

The final state definition *S_j,k_* (*j*=7, *k*=60) was selected from the hyperparameter search. Overall, 98.3% (29,270/29,765) of hospitalizations in the development set and 98.9% (6730/6805) in the test set contained at least 2 distinct states, indicating at least one transition between different states within these hospitalizations (Figure 2A). All states appeared relatively uniformly in the data with each state constituting 1.7% (SD 0.5%) of all windows on average in both the development set (3769, SD 1187; 226,178 total windows) and test set (893, SD 268; 53,591 total windows) (Figure 2B; Section B2 in Multimedia Appendix 1 for test set results).

Plots of the cluster centers show that information regarding mortality and clinicians’ actions is encoded in the states. Figure 2C and 2D indicate that a visible gradient exists in the state representation space with respect to both average mortality and average SOFA score. Figure 2E and 2F show a distinct separation in the state representation space in terms of both the clinician’s previous and next actions. Note that even without being explicitly trained for it, the state representation space captures information about the previous action.

### Evaluation of the Estimated Behavior Policy

**Figure 3.**
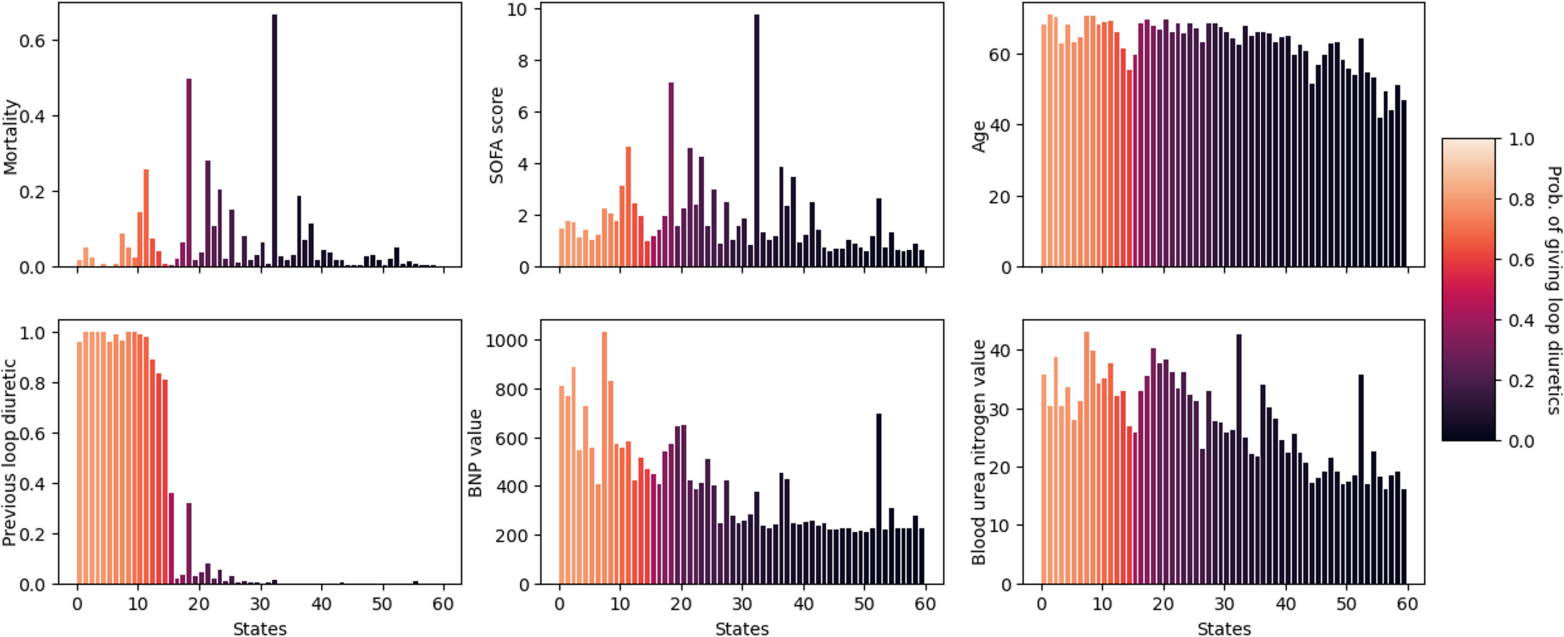
The relationship between the clinician’s likelihood of administering loop diuretics and key features. Features shown are (from left to right, top to bottom): mortality, SOFA score, age, whether loop diuretics were administered in the past 24 hours, BNP value, and blood urea nitrogen value. The height of the bars represents the average value of each feature within the state, and the color represents the clinician’s likelihood of administering loop diuretics. BNP: brain natriuretic peptide; SOFA: Sequential Organ Failure Assessment.

The estimated mortality rate of the clinician behavior policy across 1000 bootstrapped samples was 6.2% (95% CI 5.6‐6.8) (Section B9 in Multimedia Appendix 1). This was comparable to the true mortality rate of 6.2% (95% CI 5.6%‐6.8%) observed in the held-out test set, suggesting the state definitions have accurately captured clinicians’ behavior. Qualitatively, we found the estimated behavior policy to recommend loop diuretics if the patient is older, given loop diuretics the previous day, has higher brain natriuretic peptide (BNP) values, and has higher blood urea nitrogen values (Figure 3). We report trends for additional features in Section B3 in Multimedia Appendix 1.

### Evaluation of the Final Learned Policy

**Figure 4.**
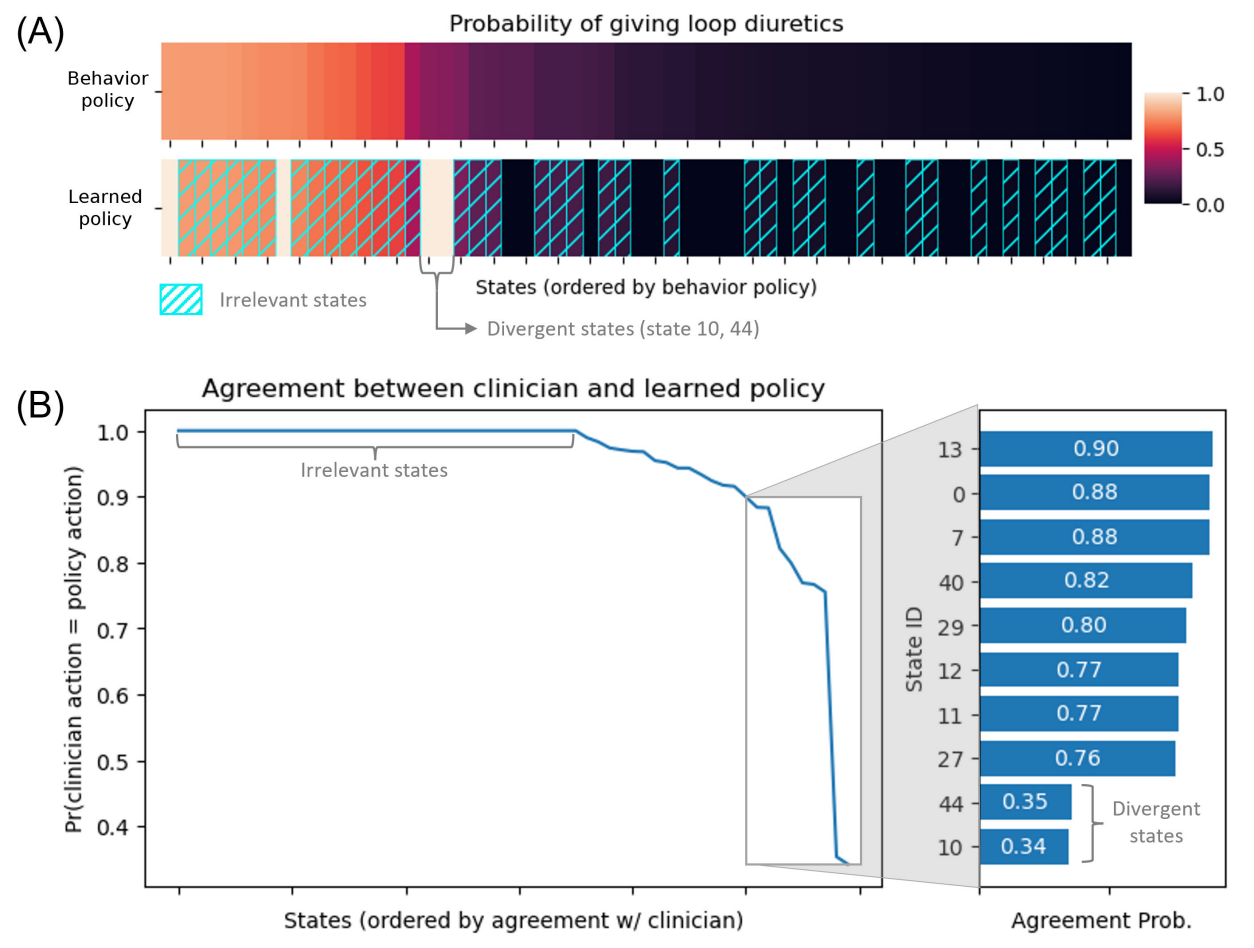
(A) Comparison of the actions recommended by the clinician behavior policy and the learned policy for each state. The color of the boxes indicates the probability of giving loop diuretics. States are ordered by decreasing likelihood of clinicians prescribing loop diuretics. Hatched boxes indicate “unimportant” states where the learned policy recommends the same actions as the behavior policy. (B) Likelihood of agreement between the clinician behavior policy and the learned policy for each state. On the left graph, states are ordered by decreasing likelihood of agreement with the clinicians. The right bar graph focuses on the 10 states where the clinicians disagree the most with the learned policy. States 10 and 44, where the likelihood of agreement is less than 0.5 (learned policy does not follow majority action), are defined as “divergent” states.

Of the 60 states, 36 were unimportant and the learned policy deferred to clinicians (Figure 4A). For the remaining 24 states, the learned policy tended to recommend the majority action: among 21,759 windows belonging to these states in the test set, only 3858 (17.7%) windows were assigned a different action under the learned policy. Yet in 2 divergent states (states 10 and 44), the learned policy did not follow the majority action. While the learned policy always recommended loop diuretics to be administered for both states, clinicians only took this action 34% (454/1326) and 35% (568/1614) of the time, respectively (Figure 4B).

On the entire held-out test set, the learned policy outperformed the behavior policy 967 times across 1000 bootstraps (96.7%) and was estimated to reduce mortality from 6.2% to 5.7%, by 0.5 (95% CI 0.0−1.1; *P*=.03) percentage points on average (Table 2). The ESS of the learned policy was 3168.45 (95% CI 3090.91-3256.65), nearly half the size of the dataset (n=6805) indicating a high confidence in the WIS estimate (Section B7 in Multimedia Appendix 1 for validation set results). On the subset of hospitalizations with divergent states, the learned policy outperformed the behavior policy 994 times across 1000 bootstraps (99.4%) and significantly decreased the estimated overall mortality from 3.8% to 2.2% by 1.6 (95% CI 0.4‐2.8; *P*=.006) percentage points on average (Table 2). The ESS of the learned policy was 550.39 (95% CI 511.49‐588.70), approximately 25% of the sample size (n=2152) and indicated a high confidence in the performance estimate. Similar improvements were observed with other OPE methods (Section B10 in Multimedia Appendix 1).

**Table 2.** Quantitative evaluation of behavior and learned policy on the held-out test set and a subset of the test set where the patient trajectories included the 2 divergent states. Values in parentheses indicate the 95% CI across 1000 bootstraps.

Dataset	Held-out test set (n=6805)		Subset with divergent states (n=2152)	
Policy	Behavior policy	Learned policy	Behavior policy	Learned policy
Estimated *J* (*π*) (95% CI)	87.56 (86.42 to 88.74)	88.59^a^ (87.10 to 90.01)	92.40 (90.89 to 93.96)	95.57^b^ (93.10 to 97.89)
Estimated improvement in *J* (*π*) (95% CI)	—^c^	1.03 (−0.05 to 2.10)	—	3.17 (0.77 to 5.46)
Estimated mortality (%) (95% CI)	6.22 (5.63 to 6.79)	5.70 (4.99 to 6.45)	3.80 (3.02 to 4.56)	2.22 (1.06 to 3.45)
Estimated decrease in mortality (%) (95% CI)	—	0.52 (−0.03 to 1.05)	—	1.58 (0.38 to 2.75)
Effective sample size (95% CI)	6805	3168.46 (3090.91 to 3256.65)	2152	550.39 (511.49 to 588.70)
% of time outperformed behavior policy	—	96.70	—	99.40
Disagreement with clinician (%) (95% CI)	22.91 (22.61 to 23.18)	21.19 (20.86 to 21.49)	30.80 (30.63 to 30.96)	32.38 (32.19 to 32.57)

a *P*=.03.

b *P*=.006.

c Not applicable.

State visualization (Section B4 in Multimedia Appendix 1) found that states 10 and 44 are close in the embedding space. Feature importance analysis of classifiers for each state showed a large overlap in key features (Section B5 in Multimedia Appendix 1), including age, previous loop diuretic, BNP value, and blood urea nitrogen value. Both states consisted of slightly older patients with an average age of 69.8 (SD 13.9) (state 10) and 68.7 (SD 13.5) (state 44) compared to the population mean of 63.4 (SD 16.2). Patients in both groups had higher BNP values (539.7, SD 960.8 vs 405.5, SD 781.8 vs 397.1, SD 687.0 for state 10, state 44, and the population, respectively) and mild kidney impairment as characterized by higher blood urea nitrogen values (35.6, SD 25.2 vs 32.9, SD 21.6 vs 28.2, SD 21.3 for state 10, state 44, and the population, respectively; Section B6 in Multimedia Appendix 1).

### Ablation Study of Pipeline

In all cases, the worst-case performance of the learned policy when one or more components were removed from the pipeline was significantly lower than the worst-case performance of the policy derived from the full pipeline (Table 3). We focus on the 2 novel aspects of the pipeline here: relaxing the unimportant states and tuning the state definitions.

**Table 3.** Worst-case performance of the learned policy when one or more of the 3 key elements in the pipeline were removed. The 3 elements are: (1) use of unimportant state relaxation (no vs yes), (2) number of data splits (single vs multiple), and (3) number of state definitions (single vs multiple). Values in parentheses indicate the 95% CI across 1000 bootstraps.^a^

Unimportant state relaxation	Number of data splits	Number of state definitions	Estimated improvement in *J* (*π*) (95% CI) (↑)	Estimated mortality % (95% CI) (↓)	% Time outperformed behavior policy (↑)
No	Single	Single	No viable policy	—^b^	—
No	Single	Multiple	No viable policy	—	—
No	Multiple	Single	No viable policy	—	—
No	Multiple	Multiple	No viable policy	—	—
Yes	Single	Single	−2.48 (−8.80 to 2.09)	7.46 (4.93 to 10.76)	20.20
Yes	Single	Multiple	−0.04 (−0.63 to 0.55)	6.24 (5.59 to 6.90)	44.90
Yes	Multiple	Single	0.45 (−1.48 to 2.22)	6.00 (4.93 to 7.18)	70.70
Yes	Multiple	Multiple	1.03 (−0.05 to 2.10)	5.70 (4.99 to 6.45)	96.70

a The estimated improvement in *J* (*π*) (↑) and estimated mortality % (↓) for the behavior policy is 0.00 (95% CI −1.14 to 1.18) and 6.22 (95% CI 5.63 to 6.79), respectively.

b Not applicable.

Removing the unimportant state relaxation led to a catastrophic failure, as no policy obtained an ESS of at least 10% the validation dataset size. This indicates overfitting, and we were unable to get a reliable estimate of the policies’ performance on the test set. Using a fixed state definition instead of tuning the state definitions led to significant variation in the performance of the learned policy depending on the data split used to learn the fixed state definition (Section B8 in Multimedia Appendix 1). In the worst-case scenario, the improvement in value of the learned policy compared to the behavior policy was −0.04 (95% CI −0.63 to 0.55), which was significantly lower than the improvement in value of 1.03 (95% CI −0.05 to 2.10; *P*=.01) of the policy derived from the full pipeline.

## Discussion

### Principal Findings

Offline RL has been applied to various health care domains [2-4]. However, a clear guide that practitioners can refer to has not been established. We present a blueprint based on previous literature to streamline the development of offline RL policies and further facilitate this through a public code base. We demonstrated the utility of our rigorous pipeline in the context of learning treatment decision policies for loop diuretics in hospitalized patients. Overall, in retrospective analysis, the learned policy was estimated to lead to significant improvement in outcome for the general patient population, especially for a subset of patients where the learned policy differed the most from clinician behavior. Though it will require prospective validation, our results reveal areas of potential improvement in current clinical care.

A key challenge in offline RL is ensuring the robustness of the learned policy. Two elements in our pipeline contributed to the improvement in robustness and performance. The first element—tuning state definitions—addresses the issue of hyperparameter sensitivity in offline RL. To select the optimal hyperparameters, prior work often relied on the hold-out method which partitioned the development dataset into training and validation sets [3,30,31]. Recently, Nie et al [7] found policy performance to be sensitive to this partitioning itself and proposed the SSR pipeline which uses multiple dataset partitions during evaluation. Building upon this insight, we show that while common practice has been to use a fixed state definition derived from a single train-validation split [3,30,31], the partitioning used to learn the state definitions can also result in significant variability of the final policy’s performance, and thus jointly tuning state definitions and policy learning over multiple data partitions is important for robustness of the learned policy.

The second element—relaxing the learned policy via unimportant states—is a form of policy constraint that mitigates the impact of extrapolation error by reducing the deviation of the learned policy from the behavior policy [5]. Using unimportant states to constrain the policy post hoc also helps reduce disruptions to the current workflow, an important consideration in health care settings. During deployment, the policy acts as an alert system to notify providers of the appropriate treatment [32]. Yet a well-known consequence in alert systems is “alert fatigue,” where providers ignore alerts due to the high frequency of irrelevant or unhelpful alerts [32,33]. By generating recommendations only when the action will meaningfully impact the outcome, unimportant state relaxation presents a simple solution to reduce disruptions to existing workflows while minimally compromising the policy’s performance.

In analyzing our learned policy, we found that loop diuretics had a limited effect on patient outcome for a sizable portion of the cohort. Our pipeline could thus be used to identify patient groups that are likely responsive to treatments. In these treatment-responsive cases, the learned policy tended to agree with the majority of clinicians, indicating that our policy could help reduce heterogeneity in treatment decisions. Patients in the 2 divergent states were slightly older and had mild kidney impairment, which could explain clinicians’ hesitancy in prescribing loop diuretics. However, the high BNP values indicate that the patients are fluid overloaded and may still benefit from diuretic treatment.

Our study is not without limitations. The pipeline used a single OPE method (WIS) during hyperparameter selection and a single dataset. While designed to be agnostic to both, future studies using external datasets and different OPE methods during hyperparameter tuning will further validate the generalizability of PROP-RL. Our problem formulation enforced decisions to be binary and to occur every 24 hours at fixed time points (Section B1 in Multimedia Appendix 1 for results across different decision points). A finer-grained problem formulation—such as specifying the exact dosage, incorporating additional actions (ie, other medications), and using shorter or more flexible time intervals for actions—along with additional data will be required to learn a policy that can be deployed in clinical settings. A promising direction for future work is incorporating clinician feedback after deployment to further refine the alert threshold and better understand when recommendations are most useful to clinicians, beyond our current approach of using unimportant states (Section A15 in Multimedia Appendix 1).

Another important limitation is our reliance on retrospective evaluation. In the absence of a reliable simulator and safety concerns associated with real-world evaluation, we relied on OPE methods which may not reflect the policy’s true performance during deployment. We mitigate this by imposing a large cutoff on the ESS during hyperparameter selection and by confirming our findings across multiple OPE methods. Nonetheless, retrospective evaluation should only be viewed as a preliminary step for identifying promising policies prior to investing in prospective studies. Future work must include robust prospective validation in accordance with guidelines such as the DECIDE-AI reporting framework [34]. The potential for unmeasured confounding is also a fundamental limitation of OPE methods. To mitigate this, we derived our state space using a comprehensive set of EHR features, selected in close consultation with a clinical collaborator with deep domain expertise. However, residual confounding may remain. Since these challenges are present in any realistic problem setting, our approach serves as a guide for other researchers to follow when learning offline RL policies.

### Conclusion

In summary, we present a standardized pipeline to streamline the development of offline RL policies in health care settings. We demonstrate the utility of this pipeline in the context of learning treatment decision policies for loop diuretics in hospitalized patients and show that the learned RL policy could potentially lead to a significant improvement in a key subset of the patient population. Our work highlights important considerations for applying RL to observational data to learn treatment decision policies, and our open-sourced code base can facilitate future development of offline RL policies on other clinical problems.

## Supplementary material

10.2196/69145 Multimedia Appendix 1 Additional methodological details and supplementary results.

10.2196/69145 Checklist 1 TRIPOD+AI checklist.

## Abbreviations

BCQ: batch-constrained Q-learning
BNP: brain natriuretic peptide
EHR: electronic health record
ESS: effective sample size
FIDDLE: Flexible Data-Driven Pipeline
HIPAA: Health Insurance Portability and Accountability Act
MDP: Markov decision process
OPE: off-policy evaluation
PCA: principal component analysis
pMDP: pessimistic Markov decision process
PROP-RL: Pipeline for Learning Robust Policies in Reinforcement Learning
RL: reinforcement learning
SOFA: Sequential Organ Failure Assessment
SSR: Split-Select-Retrain
TRIPOD+AI: Transparent Reporting of a Multivariable Prediction Model for Individual Prognosis or Diagnosis+Artificial Intelligence
WIS: weighted importance sampling
