# Supplementary material for: Optimizing Loop Diuretic Treatment for Mortality Reduction in Patients With Acute Dyspnea Using a Practical Offline Reinforcement Learning Pipeline for Health Care: Retrospective Single-Center Simulation Study
Source: JMIR Med Inform. 2025 Oct 10;13:e69145. doi: 10.2196/69145 (PMC12513688; doi:10.2196/69145)
Supplement: Multimedia Appendix 1 [file medinform-v13-e69145-s001.pdf]

Table of Contents

Section A: Methods ... 2
A1. Inclusion/exclusion criteria for cohort ... 2
A2. Data preprocessing ... 2
A3. EHR features used for state representation ... 4
A4. List of loop diuretics ... 6
A5. Training the representation model ... 7
A6. Generating the candidate state definitions ... 9
A7. Deriving key features of all states ... 10
A8. Policy learning constraints ... 10
A9. Hyperparameter selection ... 11
A10. Additional OPE methods ... 12
A11. Modifying policy for unimportant states ... 13
A12. Assumptions for WIS ... 14
A13. Calculating level of disagreement ... 15
A14. Deriving key features of divergent states ... 15
A15. Usability of the model in the context of current care ... 15
A16. Number of missing (imputed) features ... 16
Section B: Additional Results ... 21
B1. Different cut-off times for state definitions ... 21
B2. Evaluation of state definitions (test set) ... 22
B3. Visualization of behavior policy with respect to key features ... 23
B4. Visualization of states using PaCMAP ... 24
B5. Key features of divergent states ... 25
B6. Visualization of divergent states ... 26
B7. Performance of best hyperparameter across development and test sets ... 27
B8. Ablation study results ... 28
B9. Evaluation of behavior policy derived from development set on test set ... 29
B10. Evaluation of behavior and learned policy using additional OPE methods ... 30
References ... 32

Section A: Methods

A1. Inclusion/exclusion criteria for cohort

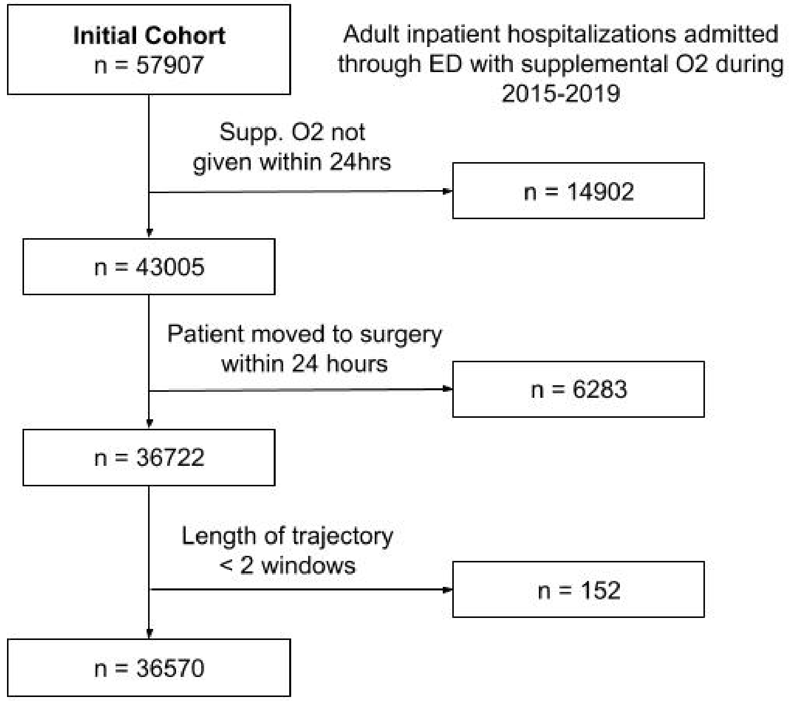

Figure S1. Flowchart of study cohort development.

A2. Data preprocessing

The first window consists of the first 4-hours of the patient's hospitalization. The second window includes this first window and ends at the following 6am. Thus, the timing of the second decision point (the end of the second window) varies depending on the time of admission. Specifically, for hospitalizations starting before 2am, the next decision point was 6am on the day of admission; for hospitalizations starting after 2am, their next decision point was 6am on the day after admission. Figure S2 shows two examples of how the hospitalization data was discretized.

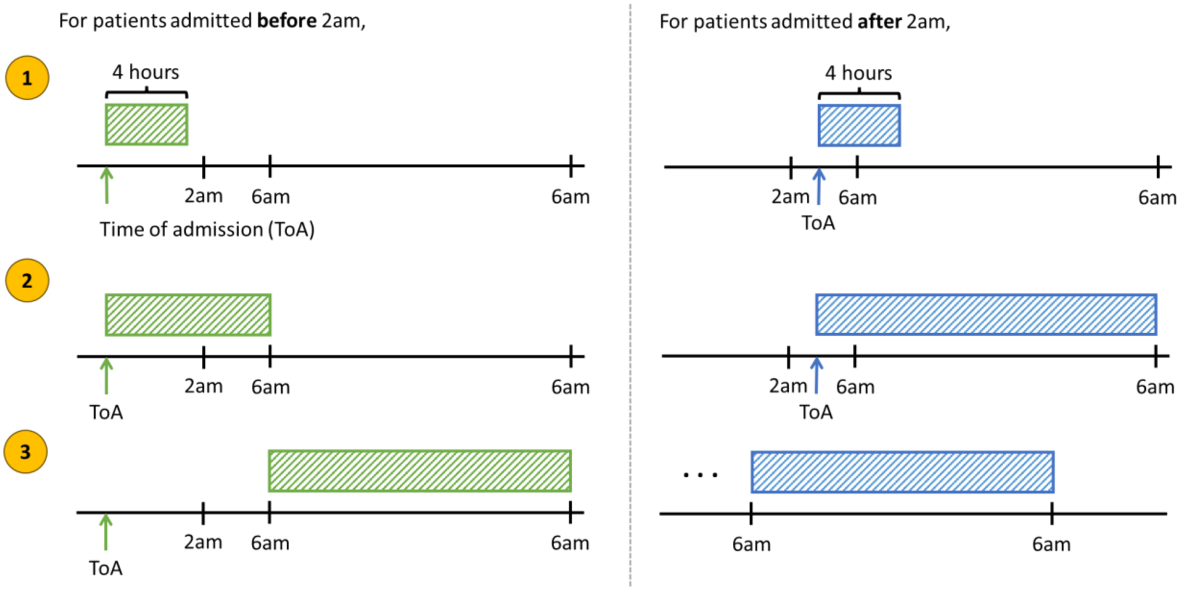

Figure S2. Windowing rules for the EHR data in cases where the patient is admitted before 2am (left) and after 2am (right). The first and second rows depict the windowing rules for the first and second windows, and the third row represents the windowing rule for subsequent windows.

The data extracted from EHR was preprocessed using the process outlined in FIDDLE [1]. Specifically, for vital sign measurements and laboratory test results, we added binary mask features to indicate whether a value was recorded in each window and a delta time feature to record the time since the previous observation. If there were multiple recordings of a variable in a given window, we used the last (i.e. most recent) recorded value within the window. If a variable was not recorded in a window (i.e., missing), we imputed its value using the "last observation carried forward" method or used the population median for windows without previous recordings (Section A16). For variables that were measured more than once in each window on average, the mean, max, and min values were also included as features. After the imputation step, each feature was normalized to have a mean of 0 and standard deviation of 1, where the normalization parameters were determined from the development set.

We formulated the problem so that the action decision (a_t) is made using observations of patient's health on day t (s_t), for treatments within day $t + 1$. This is in contrast to some prior work, where a_t is defined as treatments that occur within day t. Let's consider a scenario where we want to make a recommendation for treatments on Monday. Under the past formulation, Monday is day t and a_t includes treatments occurring on Monday. The treatment decision for Monday must be made based on s_t which is the entirety of Monday's data, and yet this recommendation would need to be available early on Monday before treatments are prescribed for the day. Thus, we will either need to wait until data collection is completed to recommend a treatment, or make a recommendation based on incomplete data. In contrast, in our formulation, Sunday is day t and Monday is day $t + 1$, a_t captures treatments that occur on Monday (day $t + 1$) and can be determined solely on s_t (data collected from Sunday). This way, we ensure that recommendations can be made on a timely manner with as much information as possible.

A3. EHR features used for state representation

Table S1. Full list of features used for creating the state representation

	Features (d=243)	Type of statistics
Demographics (1)	Age	-
Vital signs (13)	Diastolic blood pressure<br>Systolic blood pressure<br>Heart rate<br>Respiratory rate<br>Fraction of inspired oxygen (FiO2)<br>Oxygen saturation (SpO2)<br>Temperature	Binary mask<br>Last value<br>Mean, Max, Min<br>Delta time (# days since last measurement)
	Peak inspiratory pressure<br>Plateau Pressure<br>Positive end-expiratory pressure Set<br>Tidal Volume Observed<br>Tidal Volume Set<br>Tidal Volume Spontaneous	Binary mask<br>Last value<br>Delta time
Labs (46)	Alanine aminotransferase<br>Albumin<br>Alkaline phosphatase<br>Aspartate aminotransferase<br>BNP<br>Basophils<br>Bicarbonate<br>Bilirubin (conjugated)<br>Bilirubin (total)<br>Bilirubin (unconjugated)<br>Blood urea nitrogen<br>Calcium (total)<br>Calcium ionized<br>Chloride<br>Cholesterol (HDL)<br>Cholesterol (total)<br>Creatinine<br>Eosinophils (blood)<br>Fibrinogen<br>Glucose<br>Hematocrit<br>Hemoglobin<br>Lactate<br>Lactate dehydrogenase<br>Lymphocytes<br>Lymphocytes (absolute)	Binary mask<br>Last value<br>Delta time

	Magnesium<br>Mean corpuscular hemoglobin<br>Mean corpuscular hemoglobin concentration<br>Mean corpuscular volume<br>Monocytes<br>Neutrophils<br>Oxygen saturation<br>Partial pressure of carbon dioxide<br>Partial thromboplastin time<br>Phosphate<br>Platelets<br>Potassium<br>Prothrombin time<br>Red blood cell count<br>Sodium<br>Troponin-I<br>White blood cell count<br>pH<br>Partial pressure of oxygen Procalcitonin	
Meds (4)	Antibiotics<br>Anticoagulants<br>Loop diuretics<br>Steroids	Binary mask
Fluids (12)	Daily intravenous fluid input<br>Daily other fluid input<br>Daily urine output<br>Daily other fluid output<br>Daily total fluid input<br>Daily total fluid output<br>Daily total fluid balance<br>Cumulative intravenous fluid input since admission<br>Cumulative urine output since admission<br>Cumulative total fluid input since admission<br>Cumulative total fluid output since admission<br>Cumulative total fluid balance since admission	Value
SOFA scores (7)	Cardiovascular<br>Respiration<br>Central nervous system<br>Renal<br>Liver<br>Coagulation<br>Total	Last value<br>Mean, Max, Min

A4. List of loop diuretics

The following medications were defined as $a = 1$ (giving loop diuretics) in the problem setting.

Table S2. Full list of medications corresponding to the use of loop diuretics.

Medication Type	Medication Name	Route
BUMETANIDE	BUMETANIDE 0.25 MG/ML INJECTION SOLUTION	IV
	BUMETANIDE 0.5 MG TABLET	Oral
	BUMETANIDE 1 MG TABLET	Oral
	BUMETANIDE 2 MG TABLET	Oral
	BUMETANIDE INFUSION	IV
	BUMETANIDE INFUSION ADULT	IV
	BUMETANIDE INFUSION CVICU	IV
	BUMETANIDE INFUSION SYRINGE	IV
	BUMETANIDE IVPB (ADULT)	IV
ETHACRYNATE	ETHACRYNATE INJECTION	IV
ETHACRYNIC	ETHACRYNIC ACID 25 MG TABLET	Oral
FUROSEMIDE	FUROSEMIDE 10 MG (1/2 20 MG) TABLET	Oral
	FUROSEMIDE 10 MG/ML INJECTION SOLUTION	IV
	FUROSEMIDE 10 MG/ML ORAL LIQ (WRAPPER)	Oral
	FUROSEMIDE 10 MG/ML ORAL SOLUTION	IV
	FUROSEMIDE 20 MG TABLET	Oral
	FUROSEMIDE 40 MG TABLET	Oral
	FUROSEMIDE 40 MG/4 ML ORAL SOLUTION	Oral
	FUROSEMIDE 80 MG TABLET	Oral
	FUROSEMIDE INFUSION	IV
	FUROSEMIDE INFUSION - PEDS - NO DILUENT	IV
	FUROSEMIDE INFUSION CVICU	IV
	FUROSEMIDE INFUSION SYRINGE	IV
	FUROSEMIDE INJECTION SYRINGE (PEDS)	IV
	FUROSEMIDE IVPB (ADULT)	IV
TORSEMIDE	TORSEMIDE 10 MG TABLET	Oral
	TORSEMIDE 100 MG TABLET	Oral
	TORSEMIDE 20 MG TABLET	Oral
	TORSEMIDE 50 MG (1/2 100 MG) TABLET	Oral
	TORSEMIDE INFUSION	IV

A5. Training the representation model

Inspired by recent work in learning treatment effects [2], we learned a lower dimensional representation of the daily feature vector by training a multi-head deep learning model to predict the clinician's next action (treatment decision) and the hospitalization outcome given only the feature vector representation on a single day as input and the next action and patient outcome as outputs. This supervised learning task encourages a learned representation that contains information that drives clinicians' decisions, while also learning the combined effects of the state and action on the final outcome.

A multi-head deep learning model (Figure S3) was used to learn a lower dimensional representation of the daily feature vector. One head was used to predict the clinician's next action and the other head was used to predict the outcome conditioned on the next action. The base layer consists of two fully connected layers with ReLU activations. It takes the 243-dimensional feature vector as input and outputs the 32-dimensional representation. This representation is shared between the two heads, each of which consists of two fully connected layers with ReLU activations in between and a sigmoid activation at the last layer. Head 1, which predicts the clinician's next action, outputs a single value corresponding to the likelihood of the clinician administering loop diuretics. Head 2 outputs two values, each corresponding to the likelihood of death in the event the next action is 0 (loop diuretics not given) or 1 (loop diuretics given).

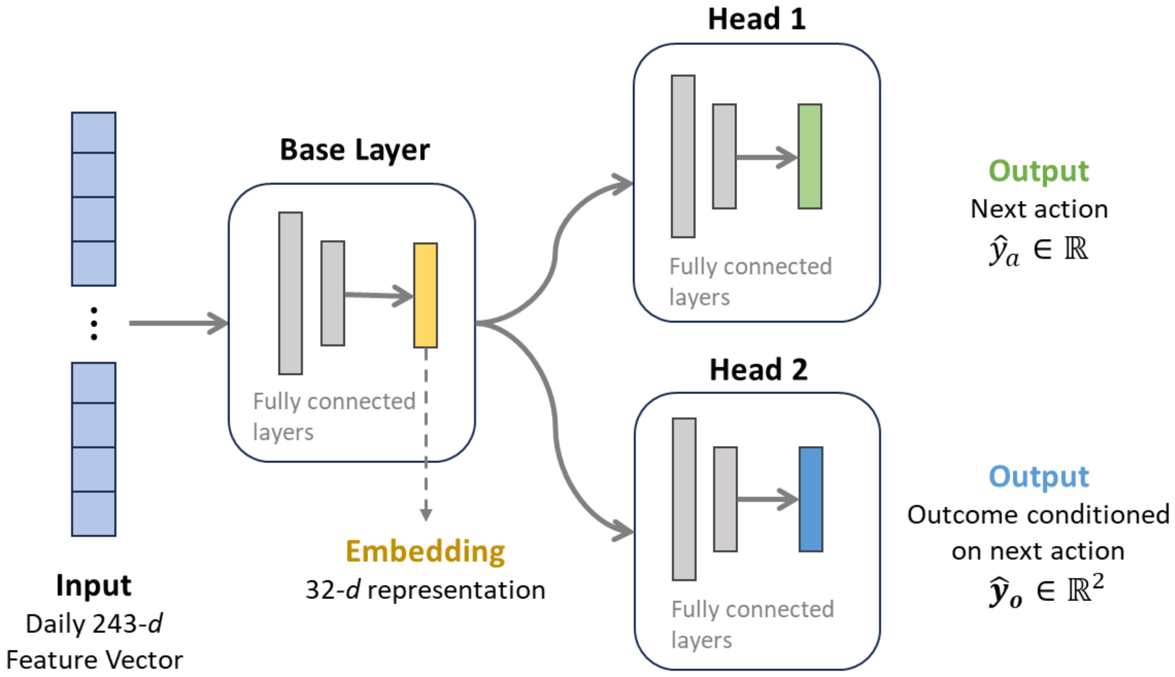

Figure S3. Diagram of representation model

Given the true next action $y_a \in \{0,1\}$ and true outcome $y_o \in \{0,1\}$, the model was trained to optimize the following loss:

$$L_{total} = L_{next\ action} + L_{mortality}$$
$$L_{next\ action} = BCE(y_a, \hat{y}_a),$$
$$L_{mortality} = BCE(\boldsymbol{y_o}, \boldsymbol{\hat{y}_o})$$
$$\boldsymbol{\hat{y}_o} = (1 - y_a)\boldsymbol{\hat{y}_o}^{(1)} + y_a \boldsymbol{\hat{y}_o}^{(2)}$$

where $L_{next\ action}$ is the binary cross entropy (BCE) loss for predicting the next action taken by the clinician and $L_{mortality}$ is the BCE loss for predicting mortality conditioned on the next action.

The model was implemented in PyTorch and trained using an Adam optimizer and tuned over a wide hyperparameter search space (Table S3). 10 unique models were trained, one for each training/validation split of the development set. Each model was trained on the training set and validated on the held-out validation set with an early stopping of 20 epochs' patience. We selected the set of hyperparameters with the best average performance (lowest validation loss) across all 10 splits. The final training hyperparameters were a learning rate of 1e-4 and batch size of 256. The final model hyperparameters were set as follows: hidden size in the base layer was set to 64, embedding size was set to 32, and the hidden size in the heads was set to 4.

**Model Hyperparameters**	
h_1: hidden size in base layer<br>l : embedding size<br>h_2: hidden size in heads	$h_1 \in \{32, 64, 128, 256\}$<br>$l \in \{8, 16, 32, 64\}$<br>$h_2 \in \{4, 8, 16, 32\}$
**Training Hyperparameters**	
μ: learning rate<br>b: batch size	$\mu \in \{10^{-2}, 10^{-3}, 10^{-4}, 10^{-5}\}$<br>$b \in \{128, 256, 512, 1024\}$

Table S3. Hyperparameter search space for representation model

A6. Generating the candidate state definitions

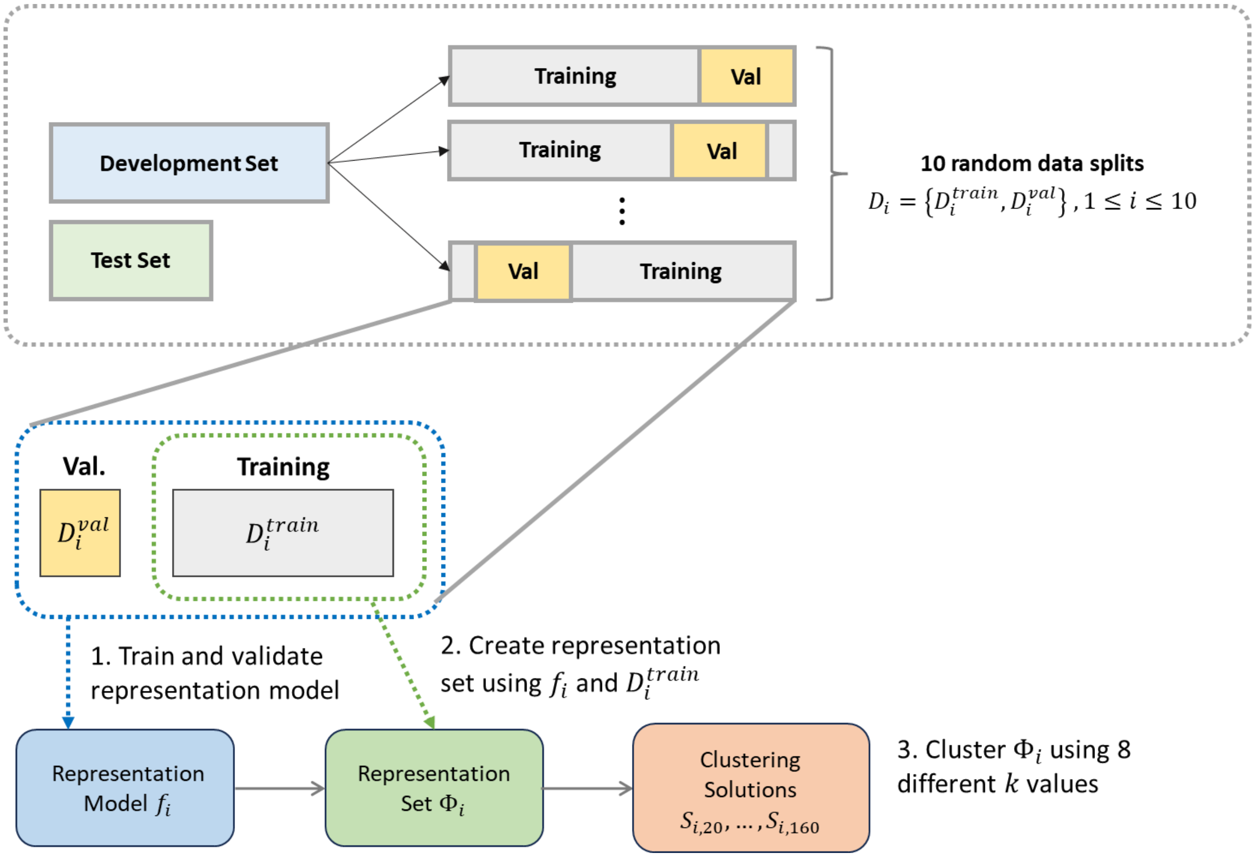

Figure S4. Diagram of state definition generation process

A total of 80 candidate state definitions were derived from 10 partitions of the development set. Figure S4 shows the generation process. The development set was partitioned into 10 training and validation splits $\{D_i{}^{train}, D_i{}^{val} \mid 1 \leq i \leq 10\}$. For each data split D_i, we learned a representation model $f_i: \mathbb{R}^{243} \rightarrow \mathbb{R}^{32}$ which was trained on $D_i{}^{train}$ and validated on $D_i{}^{val}$. f_i was applied to $D_i{}^{train}$ to create the set of representations $\Phi_i = f_i(D_i{}^{train})$. We clustered each Φ_i using ensemble k-means clustering with 8 different k values, leading to a total of 80 candidate state definitions $S_{i,k}$ where $1 \leq i \leq 10$ and $k \in \{20, 40, \cdots, 160\}$.

In ensemble k-means clustering, the data is bootstrapped (sampled with replacement) multiple times and a clustering solution is found for bootstrap. The solutions from each bootstrap are later combined to form the final clustering solution. This type of clustering method has been found to be more stable in general [3]. We applied an additional constraint when creating bootstraps so that only one window was selected from each hospitalization trajectory. This reduces the chance that the clustering algorithm will cluster all the windows within the same hospitalization (i.e., windows that will share similar features such as age) into the same cluster. 150 bootstraps were used to generate the final cluster.

A7. Deriving key features of all states

To characterize the behavior policy with respect to key features that define the states, we first trained and validated a random forecast classifier on the development set to classify all states. The classifier was implemented using the scikit-learn package and included 100 trees with a minimum of 50 samples required in the leaf node. The mean accuracy of the final classifier was 0.47 on the validation set and 0.33 on the test set. We selected the top 15 features using permutation importance (Figure S5).

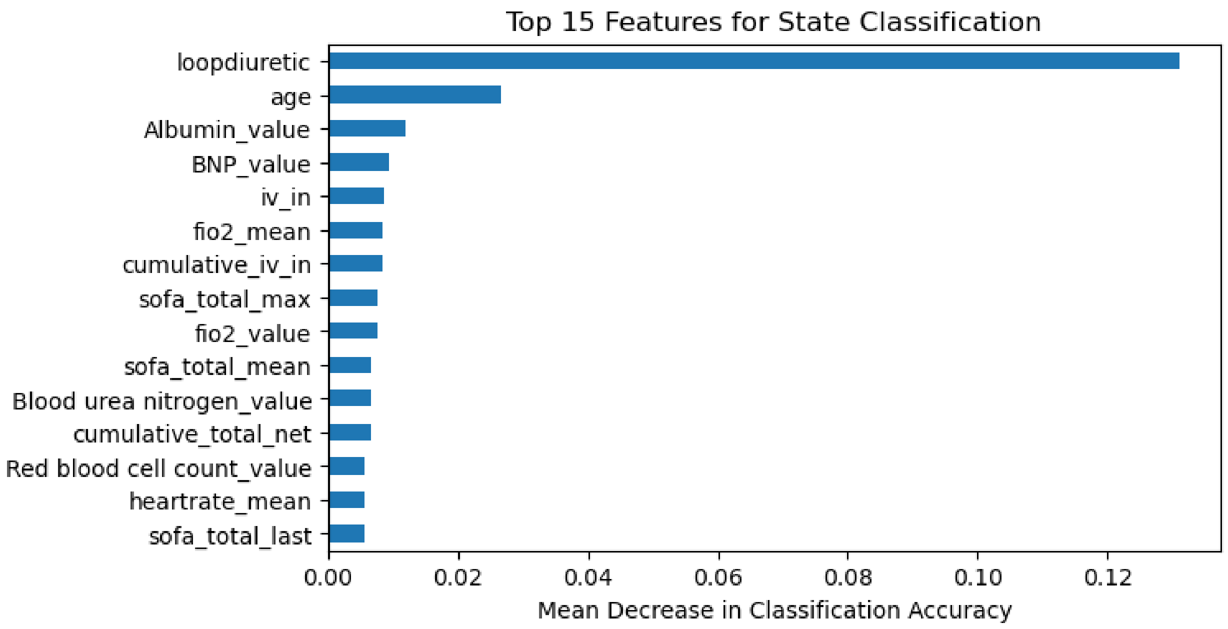

Figure S5. Top 15 important features for classifying all states based on the final state definition.

A8. Policy learning constraints

Batch Constrained Q-Learning (BCQ) [4]: BCQ aims to reduce extrapolation error by avoiding actions that were unlikely to be selected by the behavioral policy. More concretely, given a threshold $\alpha \in [0,1]$, if $\pi_b(a|s) < \alpha \max_{a \in A} \pi_b(a|s)$ we restrict our learned policy from taking action a at state s.

Pessimistic MDP (pMDP) [5]: pMDP partitions the state-action space into "known" and "unknown" regions. An ensemble of MDPs that are created from the bootstrapped data is used to determine whether we have high confidence for a given state-action transition (a.k.a. the transition probability is "known"). If, for a given state-action pair (s, a), the maximum total variation distance D_{TV} across the ensemble of MDPs is above a certain threshold θ, i.e. $D_{TV}(P_i(\cdot\ |s, a), P_j(\cdot\ |s, a)) > \theta$ where P_i and P_j are transition probabilities of the ensemble MDPs, we assume that taking action a at state s will lead to the absorbing state. To err on the side of caution, we adopt a pessimistic approach and assume that absorbing states will lead to death.

A9. Hyperparameter selection

For each data split and set of hyperparameters, the resulting policy was evaluated in terms of improvement in value compared to the behavior policy and the effective sample size (ESS) on 1000 bootstraps of the validation split. The mean and median values, along with the 95% confidence interval was calculated for both measurements.

When selecting the best hyperparameter, we first applied the following filters to the validation performance:

- Across every split, 2.5% of the effective sample size must be higher than 10% of the validation size.
- Across every split, 2.5% of the improvement in value compared to the behavior policy must be higher than -1.
- The average and median improvement in value across the 10 splits should both be above 0.

We selected the best hyperparameter with the highest median value after applying the filters and trained the final policy on the entire development set using the selected hyperparameter. Table S4 summarizes all the hyperparameters tested.

While we did not apply any additional filters aside from the ones mentioned above during hyperparameter selection, additional restrictions can be placed on the number of states k and state relevancy threshold dQ, based on the use case of the final policy. For example, if the goal is to prioritize minimal disruption to workflows, one could sweep higher values of dQ to relax the policy in more states. This effectively raises the alert threshold and limits interventions to cases where intervention would have the largest effect.

Table S4. Summary of all hyperparameters.

<table>
<tr><th colspan="3">State Definition</th></tr>
<tr><td>$S_{i,k}$: state definition</td><td colspan="2">$i \in \{1, 2, \dots, 10\}$
$k \in \{20, 40, \dots, 160\}$</td></tr>
<tr><th colspan="3">Policy Hyperparameters</th></tr>
<tr><td rowspan="3">τ: action threshold (BCQ)
θ: uncertainty threshold (pMDP)</td><td>BCQ</td><td>$\tau \in \{0.0, 0.1, 0.2, 0.3, 0.4\}$</td></tr>
<tr><td>pMDP</td><td>$\theta \in \{0.1, 0.15, 0.2, 0.3\}$</td></tr>
<tr><td>BCQ + pMDP</td><td>$\theta = 0.1;\ \tau \in \{0.1, 0.2, 0.3, 0.4\}$</td></tr>
<tr><td>dQ: state relevancy threshold</td><td colspan="2">$dQ \in \{-1, 0, 1, 5, 10, 15\}$</td></tr>
<tr><td>j: iteration number in value iteration</td><td colspan="2">$j \in \{1 \cdots max\ iter\}$</td></tr>
</table>

A10. Additional OPE methods

In addition to weighted importance sampling (WIS), the behavior and final policies were evaluated using additional three additional OPE methods: fitted Q evaluation (FQE) [6], approximate model (AM) [7], and weighted doubly robust (WDR) [8].

- Fitted Q evaluation (FQE): FQE is a model-free direct method that estimates the performance of the target policy by learning the Q-function through iterative regressions. For our experiments, FQE was run with a horizon of 50 timesteps as we found value estimates to converge by that point.
- Approximate model (AM): AM is a model-based direct method that learns an approximate model of the environment and simulates the target policy using this learned model to estimate expected returns. For our experiments, we generated 6805 trajectories – matching the size of the test set – until they reached termination under each of 1000 random seeds. Transitions and initial state distributions were estimated from the total held-out test set.
- Weighted doubly robust (WDR): WDR is a hybrid/doubly-robust method that combines aspects of importance sampling and direct methods to reduce variance and improve stability. The value functions used in WDR were estimated from FQE.

Consistent with our evaluation using weighted importance sampling (WIS), performance was evaluated across 1000 bootstraps for FQE and WDR. FQE was run with a horizon of 50 timesteps as we found value estimates to converge by that point. For AM, we aggregated the average reward at each seed level and used the one-sided permutation sampling test to calculate *P* values.

A11. Modifying policy for unimportant states

Given the set of trajectories D, where the return of each trajectory $\tau = (s_1, r_1, a_1, s_2, \cdots, a_{T-1}, s_T, r_T)$ is defined as $G(\tau) = \sum_{t=1}^{T} \gamma^{t-1} r_t$, the IS estimator is given by the weighted average

$$\hat{V}_{IS}^{\pi}(D) = \frac{\sum_{\tau \in D} G(\tau)\rho(\tau)}{|D|}$$

where the weight $\rho(\tau)$ is the product of likelihood ratios

$$\rho(\tau) = \prod_{t=1}^{T} \frac{\pi(a_t|s_t)}{\pi_b(a_t|s_t)}$$

while $\hat{V}_{IS}^{\pi}$ is an unbiased estimator of V^{π}, it typically has large variance. To reduce variance, WIS scales the weights to be between 0 and 1 and is given as

$$\hat{V}_{WIS}^{\pi}(D) = \frac{\sum_{\tau \in D} G(\tau)\rho(\tau)}{\sum_{\tau \in D} \rho(\tau)}$$

leading to a biased but consistent estimator.

Motivated to reduce the variance of IS estimators, Shen et al. [9] introduces the concept of unimportant (or "irrelevant" states), which are defined as states where the action taken has little to no effect on the trajectory's outcome. For an unimportant state s, the likelihood ratio $\frac{\pi(a|s)}{\pi_b(a|s)}$ simply adds meaningless variance to the IS estimator while having no effect on correcting the value estimate. Thus, Shen et al. shows that:

For a composite policy π' where,

- $\pi'(a \mid s) = \pi(a \mid s)$ if s is a relevant state
- $\pi'(a \mid s) = \pi_b(a \mid s)$ if s is an irrelevant state

The policy values of π and π' are equal: $J(\pi) = J(\pi')$.

Inspired by the concept of unimportant states, we "relax" the learned policy π by using π' as the final policy.

A12. Assumptions for WIS

Common assumption for WIS and other OPE methods include full support assumption and no confounding variables [7].

- Full support assumption: $\pi_e(a|x) > 0$ imples $\pi_b(a|x) > 0$. Without this, the importance weights in WIS may be undefined or infinite. In other OPE methods, insufficient support can lead OPE methods to extrapolate to state-action pairs never seen before, which can lead to extrapolation errors.
- No unmeasured confounding variables: This assumes that the return under the observed policy π is conditionally independent of the action taken, given the observed state S.

  $$R(\pi) \perp A \mid S$$

  While this is a common assumption made in many OPE methods (including WIS), this assumption is not testable from observed data and is often violated in observational datasets generated from healthcare applications [10].

To ensure there is sufficient support/coverage for WIS, we utilized two methods when training and evaluating the final policy: policy learning contraints and use of unimportant states. As outlined in Supplement A8, we used BCQ and pMDP to constrain the learned policy to avoid areas in the state-action space that were un-/less-observed by the behavior policy. In addition, the unimportant states were used to relax the final policy, effectively increasing the similiarty and coverage between the learned policy and behavior policy.

We attempted to minimize unmeasured confounders by including a comprehensive set of EHR features when constructing our state space, selected in close consultation with our clinical collaborator with deep domain expertise. However, residual confounding may still remain which could potentially bias the estimates of WIS.

A13. Calculating level of disagreement

Given a dataset D, the distribution of states within that dataset D_S, and the behavior policy of the clinicians $\pi_b(a|s)$, we defined the level of disagreement between two clinicians as:

$$\mathbb{E}_{s\sim D_S}\left[\mathbb{E}_{a_1\sim\pi_b(a|s),a_2\sim\pi_b(a|s)}[\mathbf{1}(a_1 \neq a_2)]\right]$$

This translates to the average likelihood that two randomly selected clinicians would disagree across all windows in the hospitalization data.

To measure the level of disagreement between a random clinician and the learned policy, we adapt the above equation so that the a_2 is derived from the composite policy $\pi'(a|s)$ as defined in Supplement A10. Thus, the level of disagreement between a random clinician and the learned policy is defined as:

$$\mathbb{E}_{s\sim D_S}\left[\mathbb{E}_{a_1\sim\pi_b(a|s),a_2\sim\pi'(a|s)}[\mathbf{1}(a_1 \neq a_2)]\right]$$

This translates to the average likelihood that a randomly selected clinician and the learned policy would disagree across all windows in the hospitalization data. In unimportant states, the learned policy would essentially behave as a random clinician and suggest each treatment at the same probability as the average clinician.

A14. Deriving key features of divergent states

We derived the key features of the divergent states by training two multiclass neural network classifiers to identify each of the divergent states. Each neural network took the raw feature vector as input and consisted of three fully connected linear layers with ReLU activation functions and dropout regularization. The classifiers were trained and validated on the development set. To identify the most important features, we calculated the Shapley values for each feature using the python shap library.

A15. Usability of the model in the context of current care

Our pipeline is designed to be used by offline RL researchers and practitioners. For the loop diuretics policy, input data is passively collected from the EHR and does not require manual entry from users. While no specialized technical training is required to generate policy recommendations, basic clinical expertise is necessary to interpret the treatment recommendations and make informed decisions. Users should be familiar with the clinical domain and understand that the model is intended to support, not replace, clinical judgement.

A16. Number of missing (imputed) features

For each of the 243 EHR features used for creating the state representation, we report the number of missing values and details on how it was imputed (Table S5), per the TRIPOD+AI guideline. No individual was omitted from our study cohort based on missing data (see Supplement A1 for cohort selection criteria). Missing values occur as many of the EHR features such as vital signs and lab tests are measured sporadically during a patient's stay, and not on a continuous or daily basis. EHR features are generally assumed to be missing at random (MAR). Based on prior literature, we used median and "last observation carry forward" (LOCF) methods to impute the feature values, both of which have been shown to perform reasonably well in retrospective clinical datasets and align with the guidelines described in FIDDLE [1,11,12]. LOCF is used to impute values for windows with previous recordings, and the population median is used for windows without previous recordings.

Table S5. Number of missing/imputed values for each EHR feature used for creating the state representation.

	Imputed statistics	Features (d=243)	Overall N=334,775	Development Set N=269,463	Test Set N=65,312
Demographics (1)	N/A	No missing/imputed values	0 (0.00)	0 (0.00)	0 (0.00)
Vital signs (13)	Mean, Max, Min	Diastolic blood pressure	229759 (68.63)	184005 (68.29)	45754 (70.05)
		Systolic blood pressure	229759 (68.63)	184005 (68.29)	45754 (70.05)
		Heart rate	228646 (68.30)	183059 (67.93)	45587 (69.80)
		Respiratory rate	228881 (68.37)	183278 (68.02)	45603 (69.82)
		Fraction of inspired oxygen (FiO2)	230910 (68.97)	184985 (68.65)	45925 (70.32)
		Oxygen saturation (SpO2)	228753 (68.33)	183146 (67.97)	45607 (69.83)
		Temperature	232319 (69.40)	185837 (68.97)	46482 (71.17)
	Last value<br>Delta time	Diastolic blood pressure	229759 (68.63)	184005 (68.29)	45754 (70.05)
		Systolic blood pressure	229759 (68.63)	184005 (68.29)	45754 (70.05)
		Heart rate	228646 (68.30)	183059 (67.93)	45587 (69.80)

		Respiratory rate	228881 (68.37)	183278 (68.02)	45603 (69.82)
		Fraction of inspired oxygen (FiO2)	230910 (68.97)	184985 (68.65)	45925 (70.32)
		Oxygen saturation (SpO2)	228753 (68.33)	183146 (67.97)	45607 (69.83)
		Temperature	232319 (69.40)	185837 (68.97)	46482 (71.17)
		Peak inspiratory pressure	323856 (96.74)	260671 (96.74)	63185 (96.74)
		Plateau Pressure	328678 (98.18)	264578 (98.19)	64100 (98.14)
		Positive end-expiratory pressure Set	323588 (96.66)	260437 (96.65)	63151 (96.69)
		Tidal Volume Observed	327074 (97.70)	263262 (97.70)	63812 (97.70)
		Tidal Volume Set	327192 (97.73)	263370 (97.74)	63822 (97.72)
		Tidal Volume Spontaneous	325560 (97.25)	262247 (97.32)	63313 (96.94)
Labs (46)		Alanine aminotransferase	271940 (81.23)	219268 (81.37)	52672 (80.65)
		Albumin	269570 (80.52)	217232 (80.62)	52338 (80.14)
		Alkaline phosphatase	271061 (80.97)	218540 (81.10)	52521 (80.42)
		Aspartate aminotransferase	271053 (80.97)	218528 (81.10)	52525 (80.42)
		BNP	309522 (92.46)	248889 (92.36)	60633 (92.84)
		Basophils	256835 (76.72)	205988 (76.44)	50847 (77.85)
		Bicarbonate	243103 (72.62)	194727 (72.26)	48376 (74.07)
		Bilirubin (conjugated)	326268 (97.46)	262441 (97.39)	63827 (97.73)
		Bilirubin (total)	271026 (80.96)	218511 (81.09)	52515 (80.41)
		Bilirubin (unconjugated)	333679 (99.67)	268603 (99.68)	65076 (99.64)

		Blood urea nitrogen	243017 (72.59)	194648 (72.24)	48369 (74.06)
		Calcium (total)	242735 (72.51)	194432 (72.16)	48303 (73.96)
		Calcium ionized	318063 (95.01)	255885 (94.96)	62178 (95.20)
		Chloride	242242 (72.36)	194028 (72.01)	48214 (73.82)
		Cholesterol (HDL)	332558 (99.34)	267764 (99.37)	64794 (99.21)
		Cholesterol (total)	332539 (99.33)	267749 (99.36)	64790 (99.20)
		Creatinine	242707 (72.50)	194407 (72.15)	48300 (73.95)
		Eosinophils (blood)	256848 (76.72)	205998 (76.45)	50850 (77.86)
		Fibrinogen	331917 (99.15)	267139 (99.14)	64778 (99.18)
		Glucose	242740 (72.51)	194433 (72.16)	48307 (73.96)
		Hematocrit	245972 (73.47)	196946 (73.09)	49026 (75.06)
		Hemoglobin	245085 (73.21)	196226 (72.82)	48859 (74.81)
		Lactate	275875 (82.41)	222025 (82.40)	53850 (82.45)
		Lactate dehydrogenase	330505 (98.72)	265989 (98.71)	64516 (98.78)
		Lymphocytes	256880 (76.73)	206025 (76.46)	50855 (77.86)
		Lymphocytes (absolute)	256877 (76.73)	206022 (76.46)	50855 (77.86)
		Magnesium	286947 (85.71)	231697 (85.98)	55250 (84.59)
		Mean corpuscular hemoglobin	246027 (73.49)	196992 (73.11)	49035 (75.08)
		Mean corpuscular hemoglobin concentration	246027 (73.49)	196992 (73.11)	49035 (75.08)
		Mean corpuscular volume	245972 (73.47)	196946 (73.09)	49026 (75.06)

		Monocytes	256864 (76.73)	206004 (76.45)	50860 (77.87)
		Neutrophils	256840 (76.72)	205990 (76.44)	50850 (77.86)
		Oxygen saturation	329050 (98.29)	264795 (98.27)	64255 (98.38)
		Partial pressure of carbon dioxide	322731 (96.40)	259566 (96.33)	63165 (96.71)
		Partial thromboplastin time	284229 (84.90)	228388 (84.76)	55841 (85.50)
		Phosphate	296138 (88.46)	238628 (88.56)	57510 (88.05)
		Platelets	246359 (73.59)	197293 (73.22)	49066 (75.13)
		Potassium	242086 (72.31)	193895 (71.96)	48191 (73.79)
		Prothrombin time	278052 (83.06)	223192 (82.83)	54860 (84.00)
		Red blood cell count	245972 (73.47)	196946 (73.09)	49026 (75.06)
		Sodium	242094 (72.32)	193904 (71.96)	48190 (73.78)
		Troponin-I	326726 (97.60)	261919 (97.20)	64807 (99.23)
		White blood cell count	245989 (73.48)	196974 (73.10)	49015 (75.05)
		pH	275899 (82.41)	222052 (82.41)	53847 (82.45)
		Partial pressure of oxygen	322733 (96.40)	259569 (96.33)	63164 (96.71)
		Procalcitonin	323303 (96.57)	260445 (96.65)	62858 (96.24)
Meds (4)	N/A	No missing/imputed values	0 (0.00)	0 (0.00)	0 (0.00)
Fluids (12)	N/A	No missing/imputed values	0 (0.00)	0 (0.00)	0 (0.00)
SOFA scores (7)	Last value<br>Mean, Max, Min	Cardiovascular	38517 (11.51)	25464 (9.45)	13053 (19.99)
		Respiration	38517 (11.51)	25464 (9.45)	13053 (19.99)

		Central nervous system	38517 (11.51)	25464 (9.45)	13053 (19.99)
		Renal	38517 (11.51)	25464 (9.45)	13053 (19.99)
		Liver	38517 (11.51)	25464 (9.45)	13053 (19.99)
		Coagulation	38517 (11.51)	25464 (9.45)	13053 (19.99)
		Total	38517 (11.51)	25464 (9.45)	13053 (19.99)

Section B: Additional Results

B1. Different cut-off times for state definitions

Table S6. Performance of the final policy on the entire held-out test set based on different cut-off times (6am, 8am, 10am) for the state definitions. Values in parentheses indicate the 95% confidence intervals.

**Policy cut-off time**	6am	8am	10am
Est. improvement in $J(\pi)$	1.03<br>(-0.05, 2.10)	0.16<br>(-0.46, 0.82)	-0.01<br>(-0.02, 0.01)
Est. decrease in mortality (%)	0.52<br>(-0.03, 1.05)	0.08<br>(-0.23, 0.41)	0.00<br>(0.01, 0.00)
Effective sample size	3168.46<br>(3090.91, 3256.65)	5628.65<br>(5577.38, 5676.30)	6792.38<br>(6787.93, 6796.78)
% of time outperformed behavior policy	96.70%	68.60%	14.60%
Level of disagreement with clinician (%)	21.19<br>(20.86, 21.49)	18.10<br>(18.42, 17.78)	21.29<br>(21.59, 21.00)

B2. Evaluation of state definitions (test set)

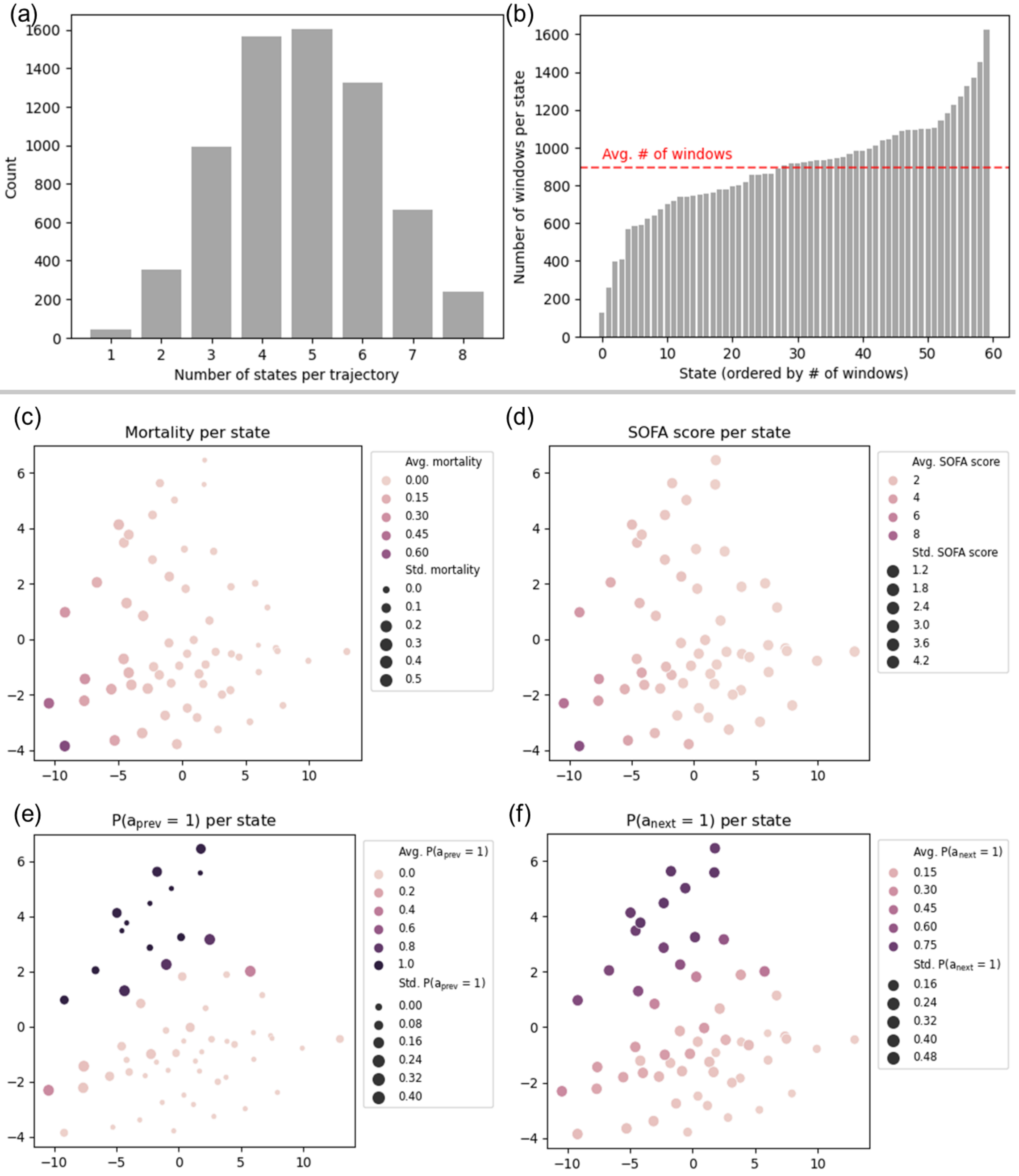

Figure S6. Sanity check of the state definitions for the test set. First row shows histograms depicting (a) number of states in each trajectory and (b) number of windows in each state. Second and third rows show PCA analysis of the representations of the cluster centers that define each state. The hue and size of each dot represents the average and standard deviation of the feature value of all samples in that state. The features are (c) mortality rate, (d) SOFA score, (e) whether loop diuretics were administered in the past 24 hours, and (f) whether clinicians chose to administer loop diuretics.

B3. Visualization of behavior policy with respect to key features

We visualized the relationship between the clinician's behavior policy (likelihood of administering loop diuretics) to key features identified in Supplement A7.

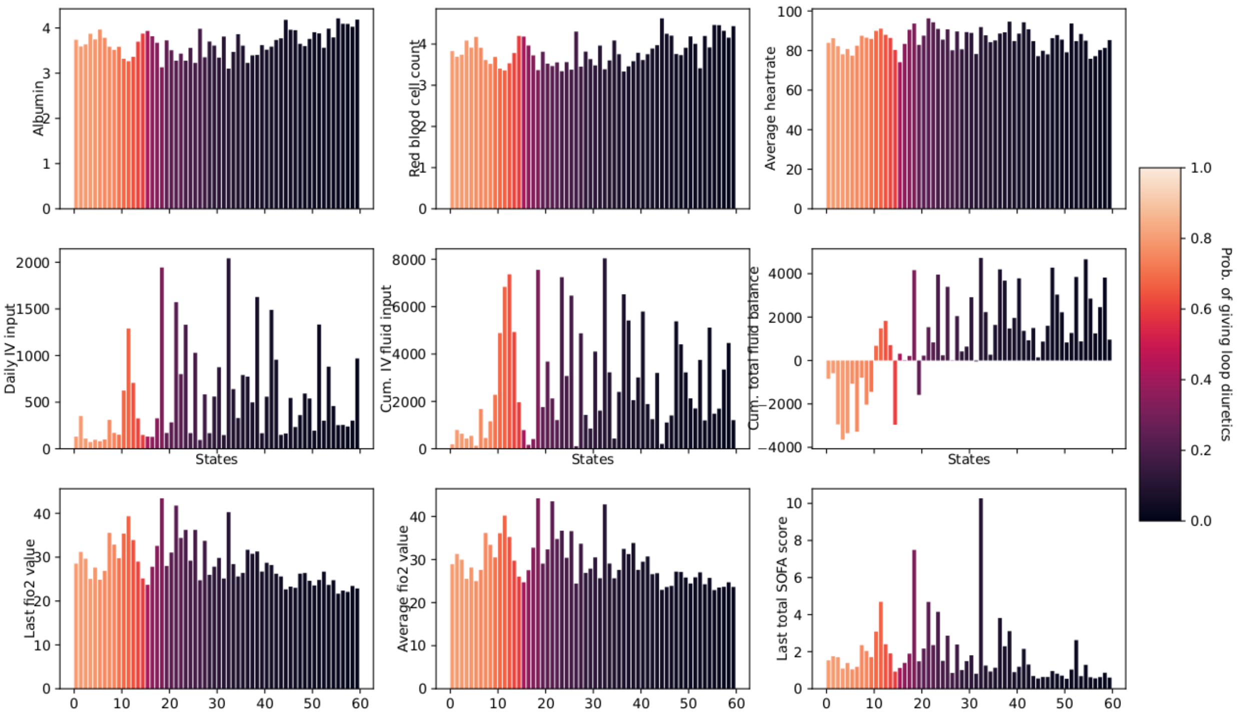

Figure S7. Relationship between clinician's likelihood of administering loop diuretics and key features. Features shown are (from left to right, top to bottom): albumin, red blood cell count, average heart rate, daily IV fluid input, cumulative IV fluid input, cumulative total fluid balance, last fio2 value, average fio2 value, and last total SOFA score. Height of the bars represent the average value of each feature within the state and color represents the clinician's likelihood of administering loop diuretics.

B4. Visualization of states using PaCMAP

We visualized all the samples using PaCMAP, a dimensionality reduction method proposed by Wang et al. [13] that focuses on preserving both local and global structures. PaCMAP visualization of all embeddings show that k-means clustering led to good separation of the states within the representation space (Figure S8).

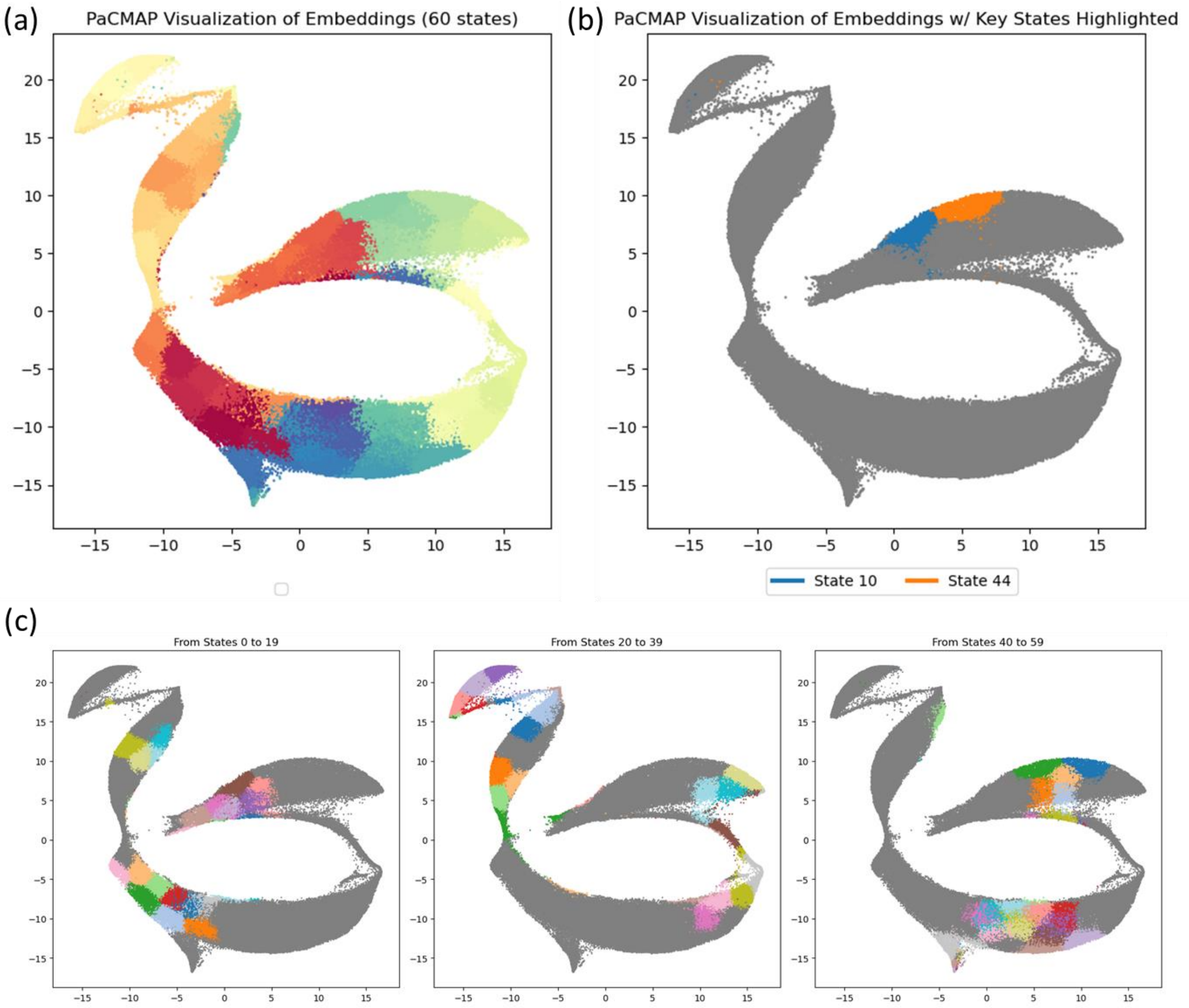

Figure S8. (a) PaCMAP visualization of the embeddings, colored by state. (b) PaCMAP visualization with the divergent states (state 10, 44) highlighted. (c) Subset of states highlighted to demonstrate the distinctness of each state and avoid overlapping colors.

B5. Key features of divergent states

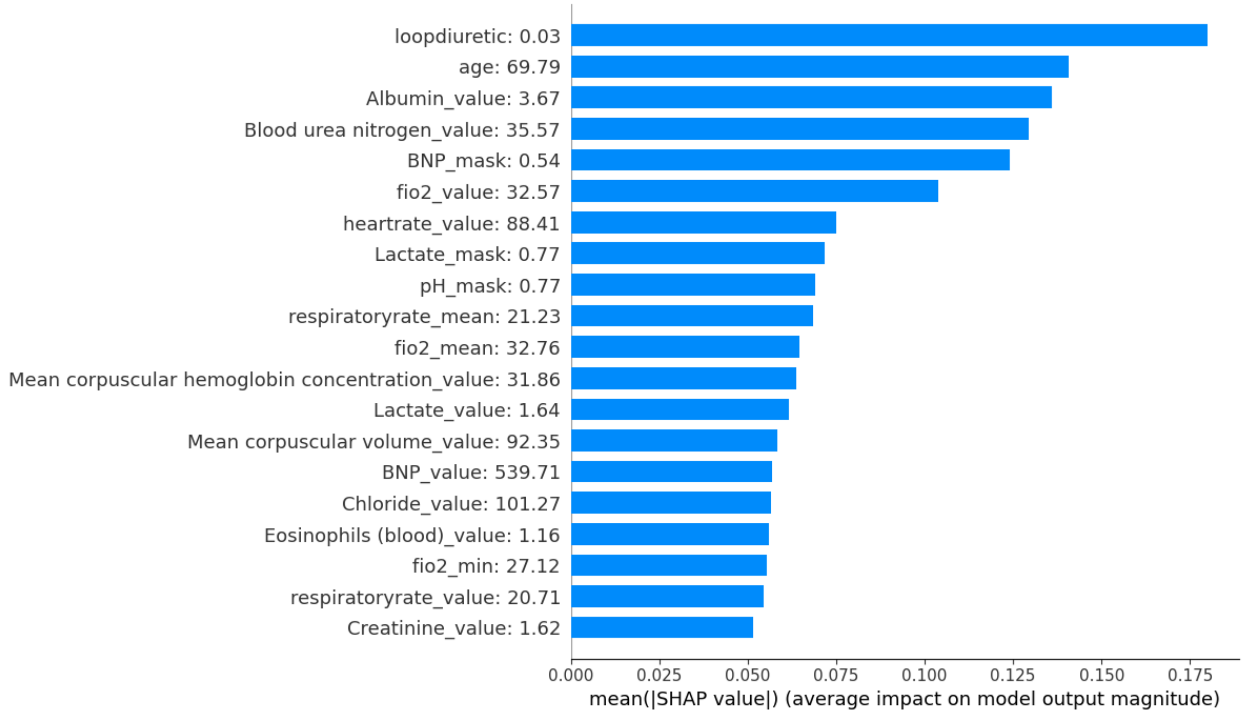

Figure S9. Top 20 features characterizing state 10 identified through Shapley values. Y axis labels show the feature name and its average value in state 10.

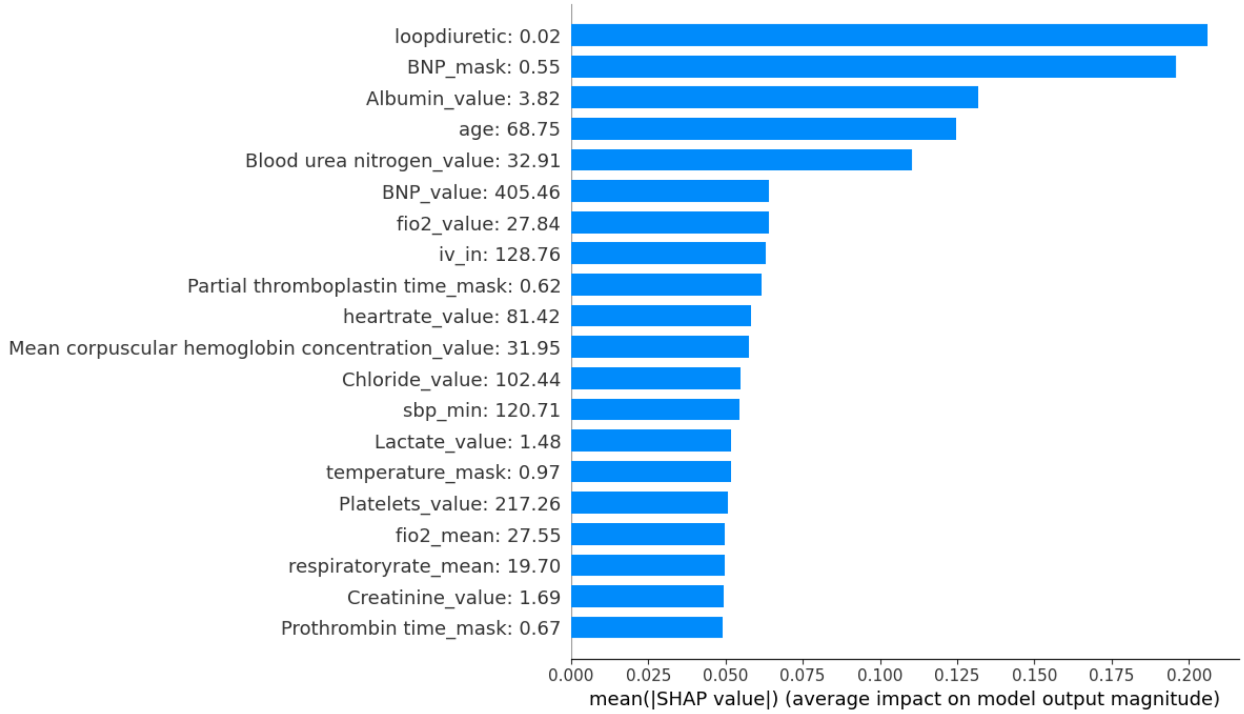

Figure S10. Top 20 features characterizing state 44 identified through Shapley values. Y axis labels show the feature name and its average value in state 44.

B6. Visualization of divergent states

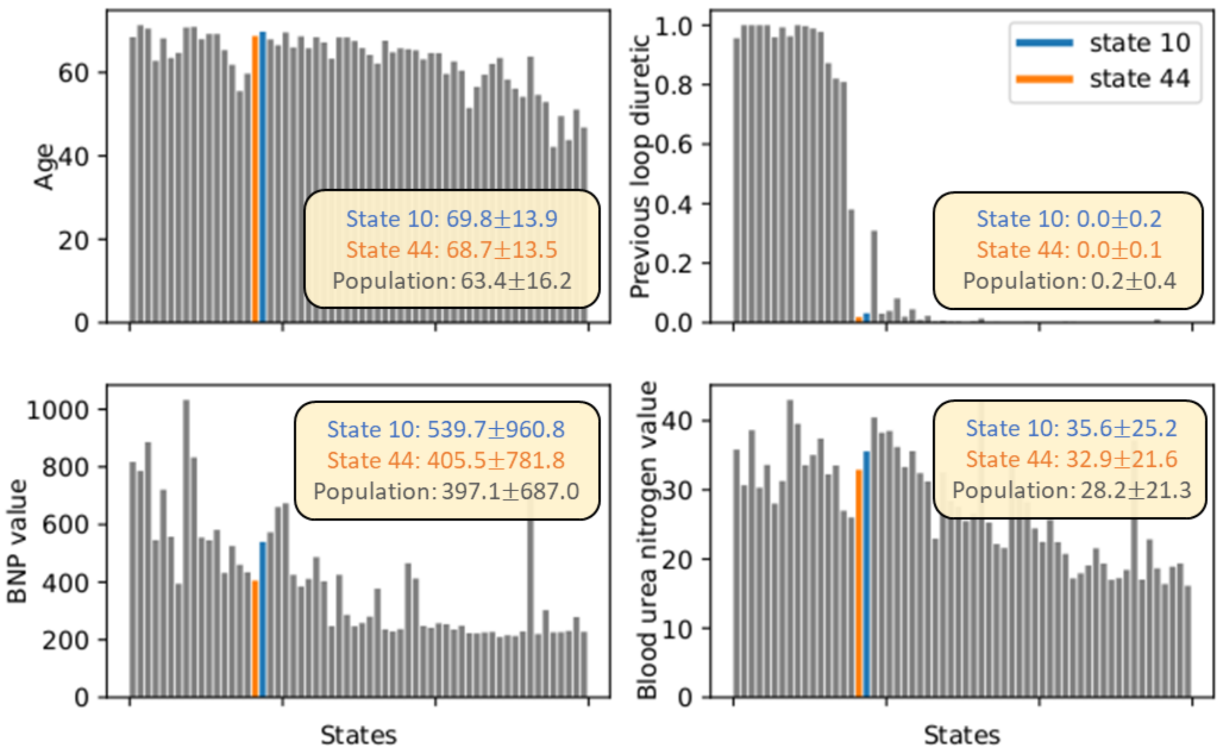

Figure S11. Average value of features (age, previous loop diuretic, BNP value, and blood urea nitrogen value) for the states. Divergent states are highlighted in blue (state 10) and orange (state 44). Values in the text box indicate the average and standard deviation of the features for the divergent states and for the overall population.

B7. Performance of best hyperparameter across development and test sets

The final policy was learned using the BCQ constraint with $\tau = 0.3$, a state relevancy threshold dQ was set to 10 and the policy resulting from a single iteration of the value iteration algorithm was selected.

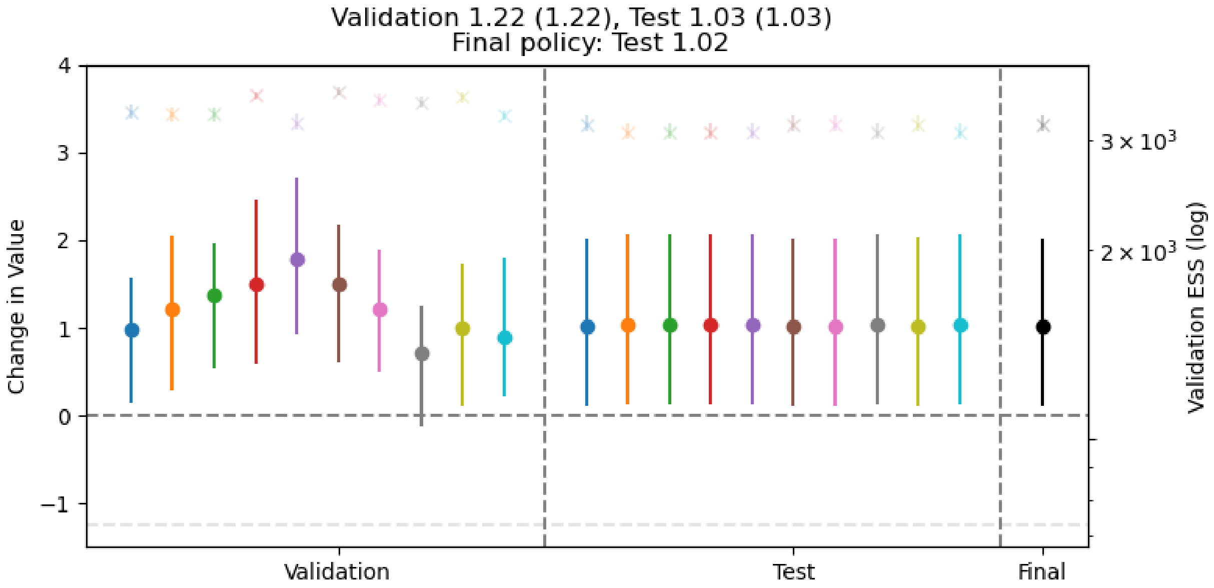

Figure S12. Performance of the best hyperparameter across the development and test sets. Leftmost panel shows the validation performance of the 10 policies trained using the best hyperparameter on each of the data splits. Middle panel shows the performance of the 10 policies on the test set. Rightmost panel shows the performance of the final policy (trained on the entire development set) on the test set. The effective sample size is shown with X markers.

B8. Ablation study results

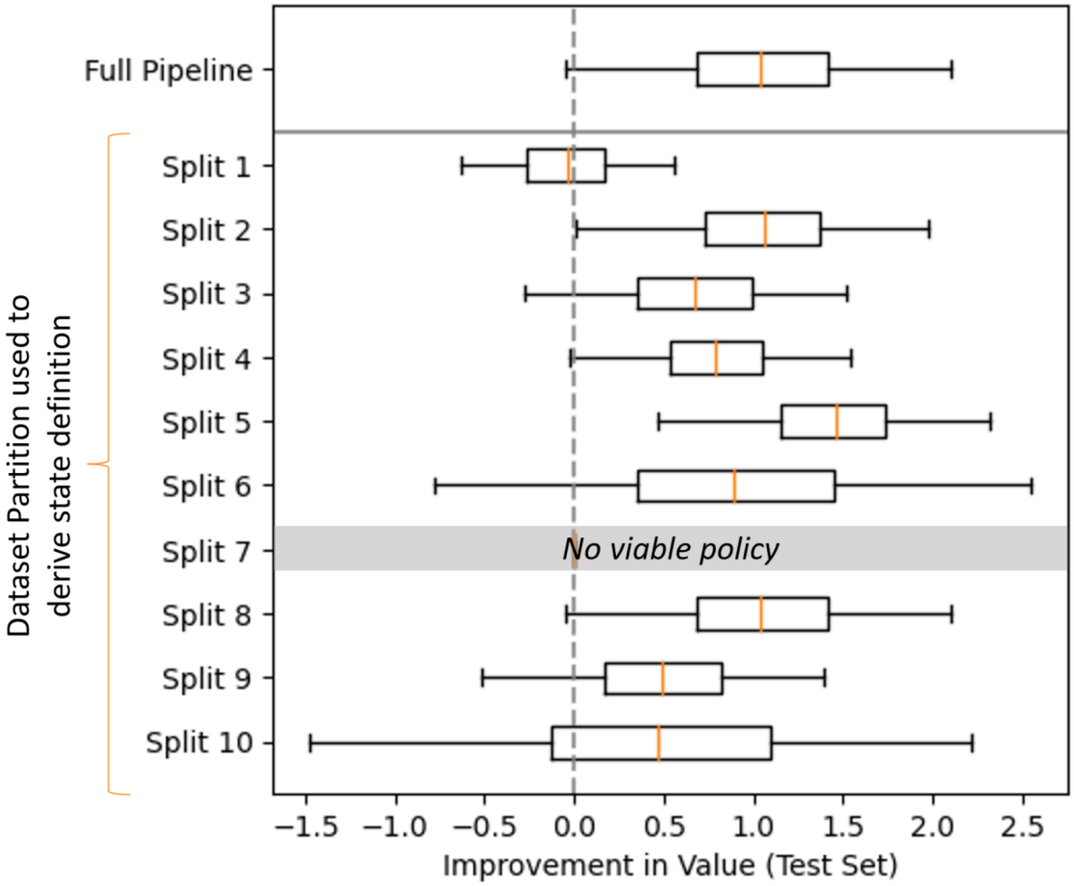

Figure S13. Estimated improvement in value (compared to the behavior policy) of the best learned policy when using a fixed state definition from a single dataset partition. Performance was measured across 1000 bootstraps. Boxes show the median and IQR and whiskers represent the 2.5% and 97.5% percentile of the performance.

B9. Evaluation of behavior policy derived from development set on test set

Table S7. Quantitative evaluation of behavior policy learned from the development set vs. behavior policy learned from the test set on the held-out test set and a subset of the test set where the patient trajectories included the two divergent states.

**Dataset**	Held-out test set (n=6805)		Subset w/ divergent states (n=2152)	
**Behavior Policy Source**	Development Set	Test Set	Development Set	Test Set
Est. $J(\pi)$	87.56 (86.42, 88.74)	87.59 (86.44, 88.79)	92.40 (90.89, 93.96)	92.42 (90.81, 93.95)
Est. mortality (%)	6.22 (5.63, 6.79)	6.20 (5.60, 6.78)	3.80 (3.02, 4.56)	3.79 (3.02, 4.59)
Effective Sample Size	6805	6683.11 (6674.88, 6690.46)	2152	2112.01 (2108.04, 2115.77)
Disagreement with Clinician (%)	22.91 (22.61, 23.18)	22.22 (21.92, 22.50)	30.80 (30.63, 30.96)	30.22 (30.05, 30.40)

B10. Evaluation of behavior and learned policy using additional OPE methods

Across all three methods – FQE, AM, and WDR – the learned policy outperformed the behavior policy in both the full held-out test set and the subset of hospitalizations including divergent states.

Table S8. Quantitative evaluation of behavior and learned policy using FQE.

**Dataset**	Test set (n=6805)		Subset w/ divergent states (n=2152)	
**Policy**	Behavior policy	Learned policy	Behavior policy	Learned policy
Est. $J(\pi)$	87.55 (86.60, 88.18)	90.70[a] (89.68, 91.66)	94.90 (91.35, 97.65)	96.02[a] (93.09, 98.31)
Est. Improvement in $J(\pi)$	..	3.14 (2.67, 3.63)	..	1.12 (0.33, 2.35)
Est. mortality (%)	6.22 (5.72, 6.70)	4.65 (4.17, 5.16)	2.55 (1.17, 4.32)	1.99 (0.84, 3.46)
Est. decrease in mortality (%)	..	1.57 (1.34, 1.82)	..	0.56 (0.17, 1.18)
% of Time Outperformed Behavior Policy	..	100.00%	..	100.00%

[a] $P < .001$;

Table S9. Quantitative evaluation of behavior and learned policy using AM.

**Dataset**	Test set (n=6805)		Subset w/ divergent states	
**Policy**	Behavior policy	Learned policy	Behavior policy	Learned policy
Est. $J(\pi)$	88.16 (87.01, 89.24)	88.78[a] (87.68, 89.83)	91.38 (89.56, 93.02)	92.94[b] (91.30, 94.46)
Est. Improvement in $J(\pi)$	..	0.62 (-0.53, 1.79)	..	1.56 (-0.80, 3.97)
Est. mortality (%)	5.92 (5.38, 6.50)	5.61 (5.08, 6.16)	4.31 (3.48, 5.22)	3.52 (2.77, 4.35)
Est. decrease in mortality (%)	..	0.31 (-0.26, 0.90)	..	0.78 (-0.40, 1.98)
% of Time Outperformed Behavior Policy	..	84.10%	..	89.90%
Sample Size	6805	6805	2011.85 (1946.00, 2082.00)	2054.83 (1984.97, 2127.00)

[a] $P = .159$; [b] $P = .101$;

Table S10. Quantitative evaluation of behavior and learned policy using WDR.

**Dataset**	Test set (n=6805)		Subset w/ divergent states (n=2152)	
**Policy**	Behavior policy	Learned policy	Behavior policy	Learned policy
Est. $J(\pi)$	87.56 (86.42, 88.74)	91.42[a] (90.05, 92.76)	92.40 (90.89, 93.96)	95.42[b] (92.77, 97.84)
Est. Improvement in $J(\pi)$	..	3.86 (2.66, 4.99)	..	3.02 (0.49, 4.56)
Est. mortality (%)	6.22 (5.63, 6.79)	4.29 (3.62, 4.98)	3.80 (3.02, 4.56)	2.29 (1.08, 3.62)
Est. decrease in mortality (%)	..	1.93 (1.33, 2.50)	..	1.51 (0.25, 2.70)
% of Time Outperformed Behavior Policy	..	100.00%	..	99.20%

[a] $P < .001$; [b] $P = .008$;

References

1 Tang S, Davarmanesh P, Song Y, *et al.* Democratizing EHR analyses with FIDDLE: a flexible data-driven preprocessing pipeline for structured clinical data. *J Am Med Inform Assoc*. 2020;27:1921–34. doi: 10.1093/jamia/ocaa139

2 Shi C, Blei DM, Veitch V. Adapting Neural Networks for the Estimation of Treatment Effects. 2019.

3 Kuncheva LI, Vetrov DP. Evaluation of Stability of k-Means Cluster Ensembles with Respect to Random Initialization. *IEEE Trans Pattern Anal Mach Intell*. 2006;28:1798–808. doi: 10.1109/TPAMI.2006.226

4 Fujimoto S, Conti E, Ghavamzadeh M, *et al.* Benchmarking Batch Deep Reinforcement Learning Algorithms. 2019.

5 Kidambi R, Rajeswaran A, Netrapalli P, *et al.* MOReL : Model-Based Offline Reinforcement Learning. 2021.

6 Le H, Voloshin C, Yue Y. Batch Policy Learning under Constraints. *Proceedings of the 36th International Conference on Machine Learning*. PMLR 2019:3703–12.

7 Voloshin C, Le HM, Jiang N, *et al.* Empirical Study of Off-Policy Policy Evaluation for Reinforcement Learning. 2021.

8 Thomas P, Brunskill E. Data-efficient off-policy policy evaluation for reinforcement learning. *International conference on machine learning*. PMLR 2016:2139–48.

9 Shen SP, Ma Y, Gottesman O, *et al.* State Relevance for Off-Policy Evaluation. *Proceedings of the 38th International Conference on Machine Learning*. PMLR 2021:9537–46.

10 Shi C, Uehara M, Huang J, *et al.* A Minimax Learning Approach to Off-Policy Evaluation in Confounded Partially Observable Markov Decision Processes.

11 Wells BJ, Chagin KM, Nowacki AS, *et al.* Strategies for Handling Missing Data in Electronic Health Record Derived Data. *eGEMs*. 2013;1:1035. doi: 10.13063/2327-9214.1035

12 Sun M, Engelhard MM, Bedoya AD, *et al.* Incorporating informatively collected laboratory data from EHR in clinical prediction models. *BMC Med Inform Decis Mak*. 2024;24:206. doi: 10.1186/s12911-024-02612-1

13 Wang Y, Huang H, Rudin C, *et al.* Understanding How Dimension Reduction Tools Work: An Empirical Approach to Deciphering t-SNE, UMAP, TriMap, and PaCMAP for Data Visualization. *J Mach Learn Res*. 2021;22:1–73.
